# Control factors and scale analysis of annual river water, sediments and carbon transport in China

**DOI:** 10.1038/srep25963

**Published:** 2016-05-11

**Authors:** Chunlin Song, Genxu Wang, Xiangyang Sun, Ruiying Chang, Tianxu Mao

**Affiliations:** 1 Institute of Mountain Hazards and Environment, Chinese Academy of Sciences, Chengdu, 610041, China; 2 University of Chinese Academy of Sciences, Beijing, 100049, China

## Abstract

Under the context of dramatic human disturbances on river system, the processes that control the transport of water, sediment, and carbon from river basins to coastal seas are not completely understood. Here we performed a quantitative synthesis for 121 sites across China to find control factors of annual river exports (Rc: runoff coefficient; TSSC: total suspended sediment concentration; TSSL: total suspended sediment loads; TOCL: total organic carbon loads) at different spatial scales. The results indicated that human activities such as dam construction and vegetation restoration might have a greater influence than climate on the transport of river sediment and carbon, although climate was a major driver of Rc. Multiple spatial scale analyses indicated that Rc increased from the small to medium scale by 20% and then decreased at the sizable scale by 20%. TSSC decreased from the small to sizeable scale but increase from the sizeable to large scales; however, TSSL significantly decreased from small (768 g·m^−2^·a^−1^) to medium spatial scale basins (258 g·m^−2^·a^−1^), and TOCL decreased from the medium to large scale. Our results will improve the understanding of water, sediment and carbon transport processes and contribute better water and land resources management strategies from different spatial scales.

The transport of water, sediments, and carbon by rivers shape the Earth’s surface, and affect the sustainable management of water and soil resources. River water, sediments and carbon transport are affected by both human activities and natural systems. The sediment and carbon contents of river water primarily originate from soil and are controlled by erosion^1^,^2^,^3^, and water discharge or lateral movement has a considerable impact on soil erosion^4^,^5^. Many environmental factors, such as climate^6^, surface runoff 7, and vegetation cover^8^, can affect the water, sediments and carbon transport processes. Topography factor like slope gradient plays important roles on runoff and erosion process that water splash and lateral movement change with slope^9^,^10^. Human activities, such as hydropower development and land conservation, may reduce or increase river sediments fluxes^11^.

Spatial scale is of central concern in hydrology and water resources science^12^,^13^, and essential for hydrological processes modelling as well as upscaling or downscaling methodologies^14^,^15^. Hydrology science has been built broadly on experiment observation such as infiltration, runoff generation, and open channel flow or soil loss at small spatial scales. With the realisation of global change, water resources responses gradually need upscaling theory from modelling and conceptualising at small space scales to large space scales. The increasing requirement for modelling need choose the appropriate models, or sets of assumptions, or equations to apply to a problem at a particular spatial scale. Model development or concept generalisation also wish mechanisms at one particular scale to be used in making predictions at other scales^16^. The need to support decision making at different spatial scales required a progressive scientific understanding of the hydrologic functioning of larger catchments^17^. Determining the mechanisms underlying hydrological processes as a function of the spatial scale is important; however, these mechanisms are difficult to identify because of the variability of hydrological processes and the heterogeneity of river basins^15^.

River sediments load on a small time scale and may be controlled by a combination of local environmental factors; however, the long-term variability may be controlled primarily by the spatial scale^18^. Previous studies have shown that small to medium rivers may exhibit a decrease in the TSS (total suspended sediment) or POC (particulate organic carbon) loads due to increased diversion and damming of many rivers and decreased impact of splash and rain-impacted flow on slopes^10^,^19^. Erode carbon decreases along with spatial scale as well^20^; however, in certain cases, the sediment yields tend to increase from smaller to larger basins since channel incision and increasing degradation of valley fill^21^,^22^. Overall, such scale effects remain controversial due to heterogeneity of river basins and limited observations. A multiple spatial scale approach has been reported to be an effective method of exploring the dynamics of river water and the transport process of sediments and carbon^23^. More integrated understanding of catchment processes from different spatial scales will compensate the poor mechanistic understanding^24^.

In recent decades, rivers across China have experienced dramatic changes related to human activities, such as rapid hydropower development^25^ and cropland conversions to forest or grassland^26^. Since the economic development and environmental protection policies in China, these activities may continue for many years. The Yangtze River and the Yellow River have experienced declines in carbon and sediment transport because of dam or reservoir construction^5^,^27^,^28^ and vegetation restoration^8^. A global level meta-analysis^23^ did not include anthropogenic factors (e.g., reservoir or dam construction, land use change), which may have significant effect on riverine transport, and did not consider a sufficient number of studies in China. In addition, studies and bulletins that contain Chinese river data are frequently published in Chinese, which limits the access to such data by the international community. Thus, the inadequate data on Chinese rivers may lead to uncertainties in their analysis. Moreover, with the dramatic human disturbances and environmental changes, the pattern of water, sediment, and carbon transport in Chinese river basins is not fully understood, particularly at different spatial scales. Therefore, a synthesis of the effects of multiple environmental drivers of river water and sediment and carbon export as well as a summary of the effects of control factors as a function of the spatial scale of the river basins across China would provide important information to help fill in these gaps. In this study, we conducted a quantitative synthesis of various data from 121 Chinese sites over a wide range of spatial scales. The objectives of this synthesis were (i) to investigate and determine the primary factors controlling the river water, sediment and carbon fluxes and (ii) to synthesize the scale effects of these environmental factors on the control of river water, sediment and carbon transport using multiple spatial scale approaches.

## Methods

### Data collection

**Figure 1:**
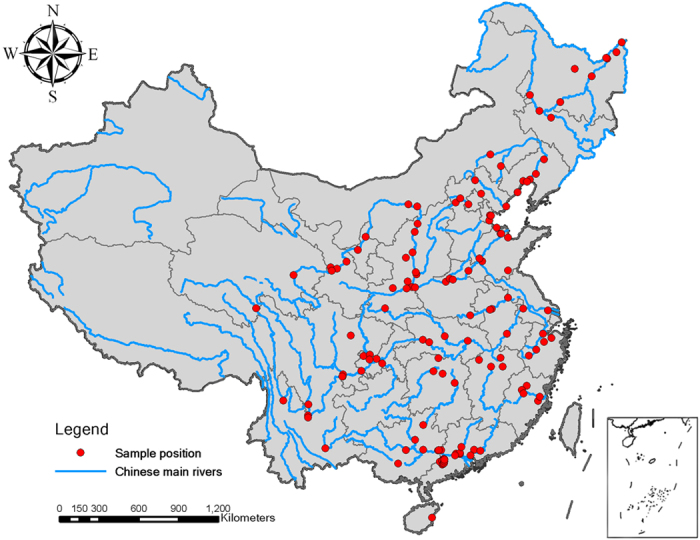
Distribution of the survey position of the basin outlet in this synthesis. The map was generated using ArcGIS for Desktop 10.0 (http://www.esri.com/software/arcgis).

The literature on the transport of water, sediment and carbon by rivers within China was reviewed. A good quality quantitative synthesis paper necessitate good quality literature searches. Our search strategy followed previous meta-analysis paper^23^. Peer-reviewed papers published before 2016 were searched according to related phrases or keywords like sediments, organic/inorganic carbon, particulate organic carbon/POC, dissolved organic carbon/DOC, particulate inorganic carbon/PIC, dissolved inorganic carbon/DIC, river/stream carbon flux. These phrases or keywords were thoroughly searched in multiple databases including the Web of Science, Google Scholar, Google, Science Direct, Springer and China Knowledge Resource Integrated Database (CNKI). In addition, we extracted river sediment and runoff data from the Chinese river sediment bulletin, which is compiled by the Chinese Ministry of Water Resources (available online: http://www.mwr.gov.cn/zwzc/hygb/). Relevant doctoral or master’s degree dissertations were also included to enlarge our database. We selected literature for analysis that satisfied the following criteria for analysis: (i) the study or survey was conducted in China, and the water samples were obtained from exoreic rivers; (ii) the characteristics of the studied rivers or basins were clearly provided or could be calculated; and (iii) at least one of the variables was provided, and the quality of the data was reliable. Any literature that didn’t meet these criteria was excluded. The raw data were obtained from the text or tables or extracted by digitizing figures using the GetData Graph Digitizer (version 2.25, Russian Federation). Based on these criteria, we selected 40 journal papers, 8 dissertations, and 9 sediment bulletins for inclusion in our database, and these references included 316 observations from 121 sites that cover all of the major basins in China (Fig. 1).

The database contained information of author(s) name, the year the literature were published, the catchment or river names, the catchment area, the location of the trials, runoff data, climate data, carbon variable(s), vegetation coverage, reservoir storage capacity index (RSCI), and soil type etc. The final compiled database included 23 control factors and carbon variables (details in Table 1). The time span for each observation varied from 1 to 61 years, with 39% of the observations occurring over the course of 1 year and 53% occurring over more than 5 years (Supplementary Figure S1). Several climate factors (e.g., MAT and MAP) were obtained from the *Atlas of Physical Geography of China*^29^ and *Chinese Water Resources Bulletin* (Chinese Ministry of Water Resources; available online: http://www.mwr.gov.cn/zwzc/hygb/) when this information was not provided in the selected literature. The MAP of the 1-year trial is equivalent to the total rainfall occurring within the trial year, and these data produced a more accurate analyses. The soil data (SOC, BD and CLAY) at the sites were obtained from the literature survey and the Harmonized World Soil Database^30^ and verified by the *China Soil Survey Data*^31^ and the *Atlas of Physical Geography of China*^29^. The total annual discharge was used to calculate runoff depth (RD). Similarly, the annual precipitation was used to calculate the runoff coefficient (Rc) when it was not provided. The reservoir storage capacity index (RSCI) is defined as the percentage of the total capacity of the reservoir divided by the annual average water discharge^27^. The reservoir storage capacity data obtained from the *China Water Conservancy Yearbook*^32^ when the information was not included in the selected literature.

### River exports variables

The river exports variables that we focused in this paper are runoff coefficient, total suspended sediment concentration, total suspended sediment load, and total organic carbon load. The runoff coefficient (Rc) is a coefficient relating the amount of surface runoff. When it was not provided by a paper, we computed Rc with runoff depth (RD) divided by annual rainfall. The total suspended sediment load refers to the value of unit area per year; when not provided, this value was calculated as:


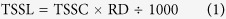


where TSSL is the estimated total suspended sediment load of unit area (g·m^−2^·a^−1^), TSSC is the Total suspended sediment concentration (mg L^−1^), RD is the runoff depth (mm).

The total organic carbon load (TOCL) was computed as follow when not provided in the literature:


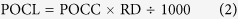



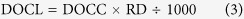



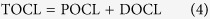


where POCL is the particulate organic carbon yield of unit area (g·m^−2^·a^−1^), POCC is the particulate organic carbon concentration (mg L^−1^), DOCL is the dissolved organic carbon yield of unit area (g·m^−2^·a^−1^), DOCC is the dissolved organic carbon concentration (mg L^−1^), RD is the runoff depth (mm), TOCL is total organic carbon load of unit area (g·m^−2^·a^−1^).

### Dataset analyses

For the synthesis, the catchment area was used as an indicator for the river basin processes related to scale effects^33^. Therefore, the first step in the analysis was to classify the basin spatial scale based on the basin area used to answer the scale effects question. Five size classes were included: small, medium, large, sizeable and great (Table 2). The environmental variables were classified according to Mutema’s principle^23^ using a variety of classes (Table 2). The sample size for each environmental factor of the different classes are listed in Table 2 and Supplementary Table S1. Summary statistics of the variables (Rc, TSSC, TSSL, and TOCL) for the different environmental factor classes were calculated to determine the variability of the water and carbon contents.

**Figure 2:**
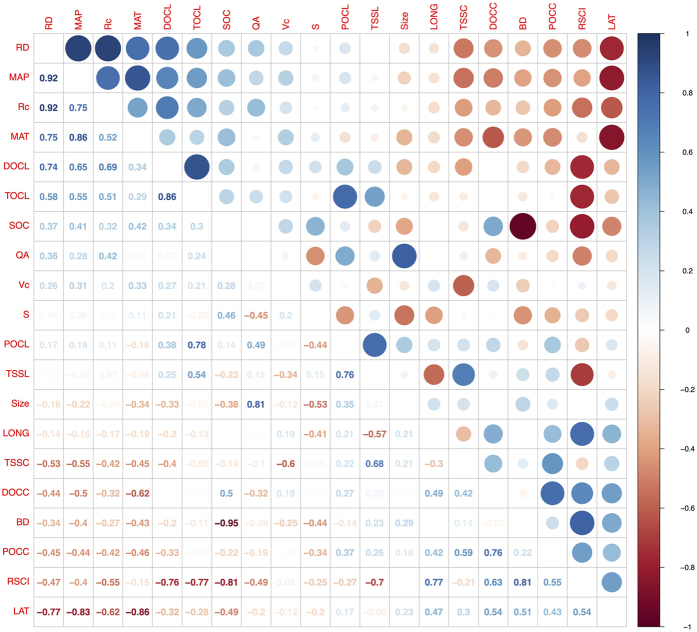
Correlation matrix of variables. Every correlation coefficient which match two variables was calculated with spearman method in R. Abbreviations of the variables as shown in Table 1. Numbers range from −1 to 1 are Spearman’s rank correlation coefficients of variables on horizontal and vertical axes. Colour depth and size of the circles indicate the correlation strength.

The second analysis step was to perform a Spearman’s rank correlation for all of the carbon variables and control factors. The environmental control factors and carbon variables typically have a monotonic nonlinear relationship^23^,^34^. Therefore, Spearman’s rank correlation was selected for our bivariate analysis. A correlation matrix (Fig. 2) with correlation coefficients was constructed using the R platform^35^ and the “corrplot” package.

**Figure 3:**
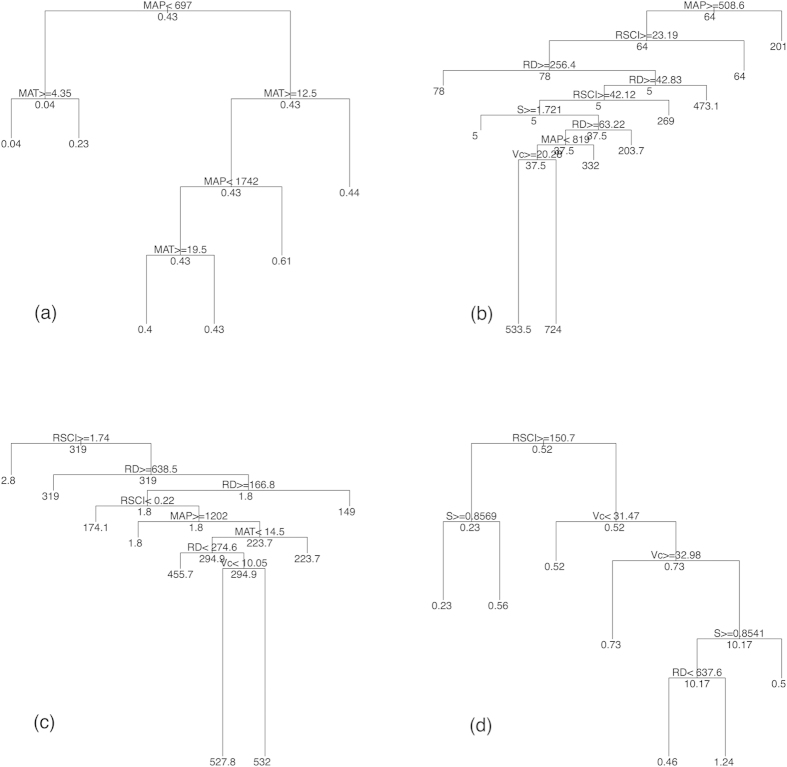
CART based regression tree of environmental variables. Splits are determined by their contribution to the overall model and reduce predictive error. The longer the branches in the tree, the greater the deviance explained. (**a**) Rc as response variable; (**b**) TSSC as response variable; (**c**) TSSL as response variable; (**d**) TOCL as response variable. Abbreviations of the variables as shows in Table 1.

The purpose of the third step was to identify the primary control factors for carbon transport within the database. We used a classification and regression tree (CART) model as an exploratory technique to identify natural and anthropogenic factors that significantly affected the carbon transport. CART is based on recursive, binary, portioning methods to determine relationships with the response variables by recursively splitting data into increasingly homogeneous subgroups with a specific CP value^36^. The CP value is a complex parameter; splits that decreases the overall lack of fit by a factor of CP is attempted. Any split that does not improve the fit by CP will likely be pruned off by cross-validation so the program need not pursue this split^35^. This process continues until either no improvement is observed or the subgroups reach a minimum size. As a tree-based hierarchical model, CART is an ideal analytical tool for exploring multivariate responses in complex data^37^ because CART can capture the interaction effects among the control factors and identify the most effective variables. We selected Rc, TSSC, TSSL, and TOCL as the response variables, and all of the relevant environmental variables were included in the model. Initially, we set the CP value to 0.001 in the model procedure and obtained the initial trees (Figures S2~S5). But these trees were too complex which needed to be pruned. Thus we used “printcp”, “plotcp”, and “prune” function of the “rpart” package to examine and regenerate the results. Finally the optimal trees were obtained with CP values of 0.012, 0.0082, 0.0079, and 0.013 for the Rc, TSSC, TSSL, and TOCL, respectively (Fig. 3, Figures S2~S5). The environmental variables selected by the CART model were used to fit multiple linear regression models. The above CART and multiple linear regression approaches were completed in R^35^ using the “rpart” and “base” package.

**Figure 4:**
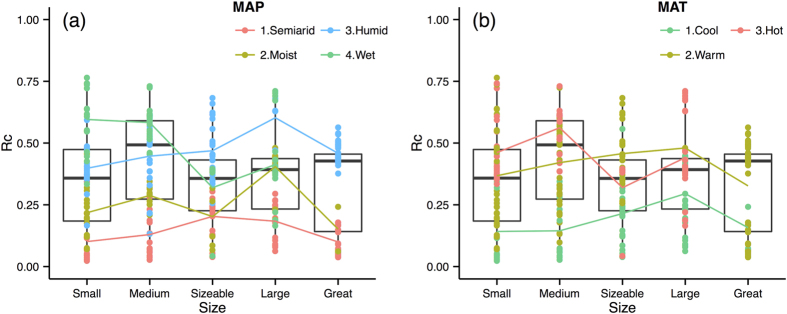
Runoff coefficient (Rc) variations along scale under different classes of environmental factors. Boxes are the 25th and 75th percentiles quantiles. Small: <15000 km^2^; Medium: 15000–100000 km^2^; Sizeable: 100000–350000 km^2^; Large: 350000–700000 km^2^; Great: >700000 km^2^. Coloured dots and lines show Rc under the different classes of averment factors change with spatial scales. Abbreviations of the variables as shows in Table 1. Classifications criteria as shows in Table 2.

**Figure 5:**
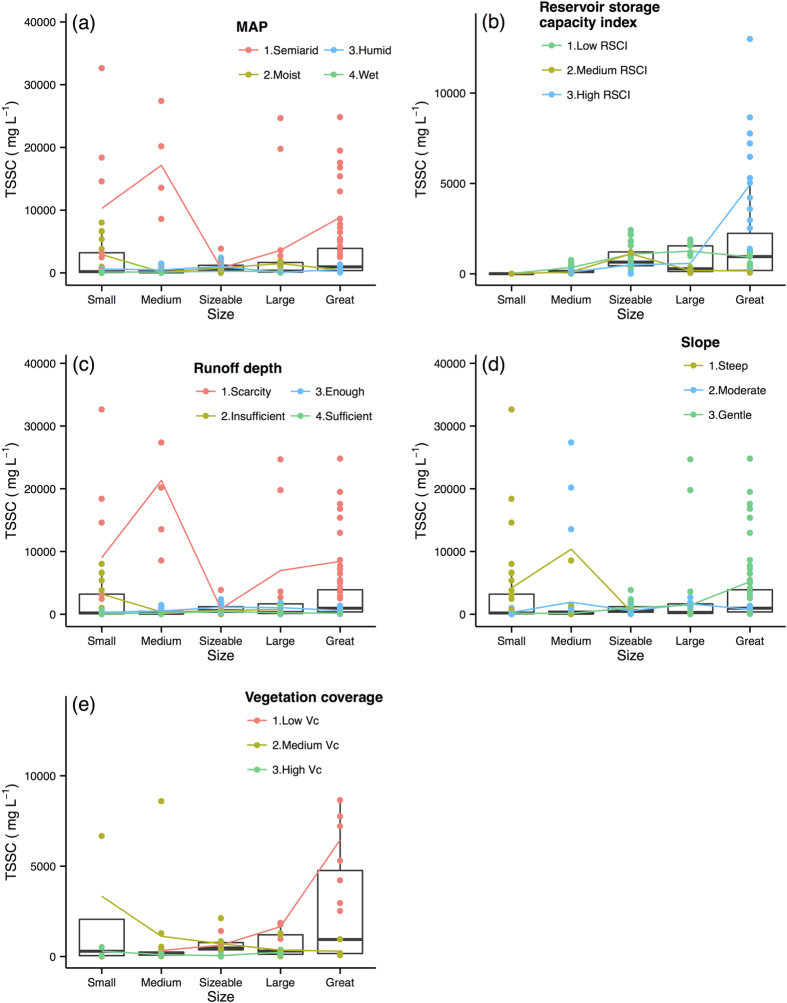
Total suspended sediments concentration (TSSC) variations along scale under different classes of environmental factors. Boxes are the 25th and 75th percentiles quantiles. Small: <15000 km^2^; Medium: 15000–100000 km^2^; Sizeable: 100000–350000 km^2^; Large: 350000–700000 km^2^; Great: >700000 km^2^. Coloured dots and lines show TSSC under the different classes of averment factors change with spatial scales. Abbreviations of the variables as shows in Table 1. Classifications criteria as shows in Table 2.

**Figure 6:**
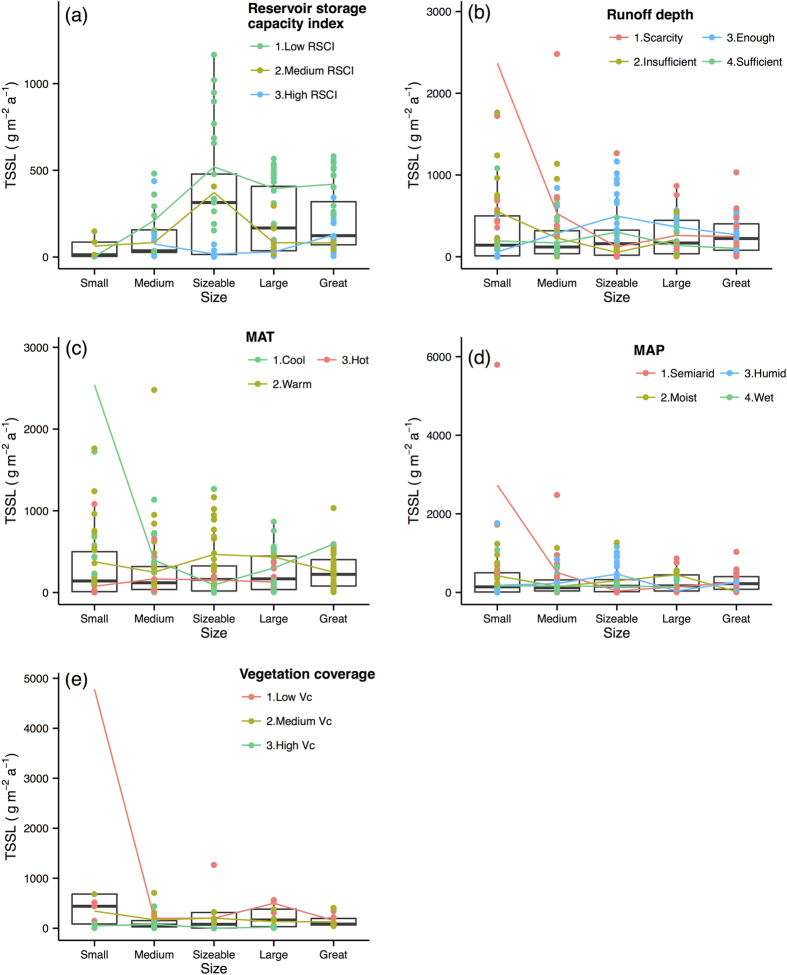
Total suspended sediments load (TSSL) variations along scale under different classes of environmental factors. Boxes are the 25th and 75th percentiles quantiles. Small: <15000 km^2^; Medium: 15000–100000 km^2^; Sizeable: 100000–350000 km^2^; Large: 350000–700000 km^2^; Great: >700000 km^2^. Coloured dots and lines show TSSL under the different classes of averment factors change with spatial scales. Abbreviations of the variables as shows in Table 1. Classifications criteria as shows in Table 2.

**Figure 7:**
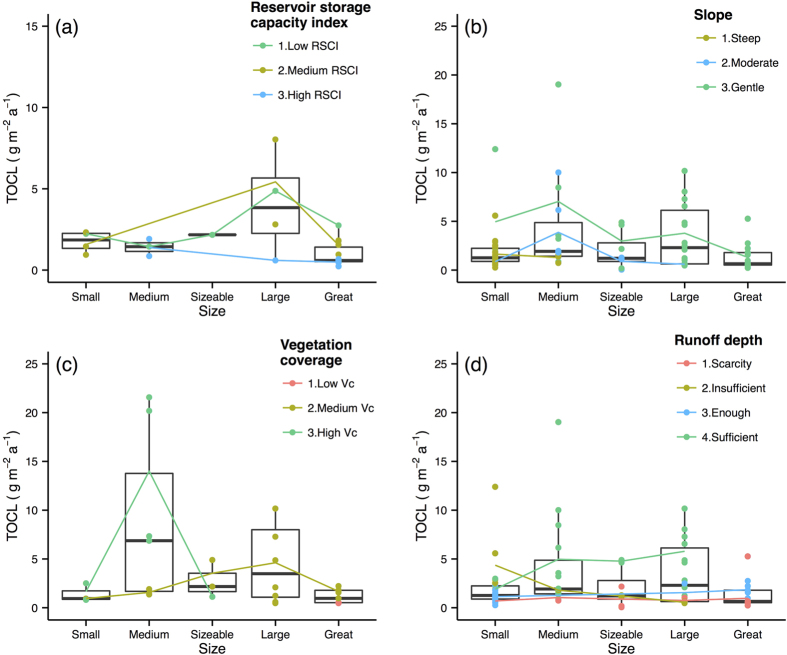
Total organic carbon load (TOCL) variations along scale under different classes of environmental factors. Boxes are the 25th and 75th percentiles quantiles. Small: <15000 km^2^; Medium: 15000–100000 km^2^; Sizeable: 100000–350000 km^2^; Large: 350000–700000 km^2^; Great: >700000 km^2^. Coloured dots and lines show TOCL under the different classes of averment factors change with spatial scales. Abbreviations of the variables as shows in Table 1. Classifications criteria as shows in Table 2.

The last analysis step was to perform a more comprehensive analysis to summarize the spatial scale effects of the selected primary control factors on the response variables. The effect of the primary control factors on the Rc, TSSC, TSSL, and TOCL are illustrated with box and average line plots (Figures 4, 5, 6, 7). Each box plot includes a scatterplot of carbon or water variables as environmental control factors, with different classes and trend lines included for each spatial scale. The box plots show that multiple control factors had scale effects on each class as well as the Rc, TSSC, TSSL, and TOCL. The inorganic carbon variables were not included because of data limitations. We used the “ggplot2” package in R^35^ to plot these graphics.

## Results

### Correlation analysis with environmental factors

The effects of the environmental factors on water, sediment, and carbon variables were estimated using Spearman’s rank correlation (Fig. 2). The results show that the RSCI had a significant negative relationship with the Rc, TSSC, TSSL, and TOCL, whereas the RSCI had a significant and positive relationship with POCC and DOCC and presented Spearman’s correlation coefficients (r_s_) of 0.55 and 0.63, respectively. The vegetation coverage (Vc) was negatively related to the TSSC and TSSL but positively related to the TOCL and Rc. The organic carbon variables (i.e., POC and POC) and TSS were strongly and positively correlated. The runoff depth (RD) increased the TOCL, with an r_s_ of 0.65, whereas it decreased the TSSC, with an r_s_ of −0.53, and the mean annual precipitation (MAP) had a similar effect on the TOCL and the TSSC. The Rc was significantly and positively correlated with the mean annual temperature (MAT) and MAP.

### Primary control factors

All of the environmental factors were considered in the CART model as predictors, whereas the Rc, TSSC, TSSL, and TOCL were the response variables. After the trial runs, we obtained the optimal trees with CP values of 0.012, 0.0082, 0.0079, and 0.013 for the Rc, TSSC, TSSL, and TOCL, respectively (Fig. 3). The results indicated that two environmental variables (MAP and MAT) provided the greatest contributions to the Rc model; five environmental variables (MAP, RSCI, RD: runoff depth, S: slope and Vc) provided the greatest contributions to the TSSC model; five environmental variables (RSCI, RD, MAP, MAT, and Vc) provided the greatest contributions to the TSSL model; and four environmental variables (RSCI, S, Vc, and RD) provided the greatest contributions to the TOCL model. Therefore, the environmental variables that provided the greatest contributions were selected as the primary control factors for the Rc, TSSC, TSSL, and TOCL. Subsequently, the Rc, TSSC, TSSL, and TOCL variables were selected as the primary control factors, and a multiple linear regression model was fit for each response variable. The model results are presented in Table 3 and Supplementary Figure S2, which indicate that the four models for the Rc, TSSC, TSSL, and TOCL were robust.

### Scale effects of water and carbon with control factors

#### Runoff coefficients (Rc)

Figure 4 shows the effects of the primary environmental factors (i.e., MAP and MAT) on the Rc according to the spatial scale. The Rc increased with the MAP and MAT for the majority of spatial scales, which is consistent with the Spearman correlation results in Fig. 2. The Rc increased from the small to large scale and then decreased at the great scale except in wet (MAP more than 1500 mm), semiarid (RD less than 600 mm but above 250 mm), and hot regions (MAT above 20 degrees). By combining the MAP and MAT classes, the mean Rc value increased by 20% from the small to medium scale and then decreased by 20% at the sizable scale and increased slightly at the great scale.

#### Total suspended sediments concentrations (TSSC)

The TSSC decreased from the medium scale to the sizeable scale and then increased to the great scale in semiarid basins (Fig. 5a) and for RDs classified as scarcity (Fig. 5c), although the data range of medium scale to the sizeable scale are similar since limited data. The RSCI appeared to have no effect on the TSSC in the small-scale basins, whereas at large scales, the TSSC and RSCI increased together (Fig. 5b). Figure 5c shows that the TSSC in the studied Chinese rivers was high for RDs classified as scarcity and for semiarid basins except for in sizeable-scale basins. TSSC generally decreased along with the MAP and RD. These results were expected because the RD and MAP have r_s_ values of −0.53 and −0.55 with TSSC, respectively (Fig. 2). The high Vc river basins had a lower TSSC at all scales, which was consistent with the r_s_ of −0.6 between the TSSC and Vc (Fig. 2); however, the low Vc river basins had a higher TSSC at the large scale (Fig. 5e). The relationship between the slope and TSSC was indistinct in Fig. 5d. Overall, TSSC decreased from the small to sizeable scale but increase from the sizeable to large scales.

#### Total suspended sediment loads (TSSL)

Considering all of the spatial scales, the TSSL generally decreased from the small to the large scale (Fig. 6), particularly from the small-scale (768 g·m^−2^·a^−1^) to the medium-scale basins (258 g·m^−2^·a^−1^). Figure 6a shows that the low RSCI rivers had a higher TSSL for all spatial scales and presented an increasing tendency along spatial scales. This pattern is consistent with the r_s_ of −0.7 between the RSCI and TSSL (Fig. 2). The TSSL in the insufficient and scarcity RD basins decreased from the small scale to the sizeable scale as shown in Fig. 6b. The hot basins exhibited the lowest TSSL at the majority of spatial scales, whereas the TSSL in the cool basins decreased drastically from the small scale to the sizeable scale (Fig. 6c). The semiarid river basins exhibited a higher TSSL, particularly at the smallest scale, and this tendency decreased along with the spatial scale, which was indicated by the substantial decrease in the TSSL of the semiarid rivers from the small to sizeable scale (Fig. 6d). Overall, the rivers with a low Vc exhibited a higher TSSL, whereas the basins with a low Vc exhibited decreasing TSSL values from the small to medium scale and then increasing values from the sizeable to large scale (Fig. 6e). The median value of TSSL was observed to decrease from the small scale to the medium scale by 16% and then increase from the medium scale to the great scale by 87%.

#### Total organic carbon loads (TOCL)

The high RSCI basins had lower TOCL at all scales, whereas the TOCL increased in basins with low and medium RSCI from the small scale to the large scale (Fig. 7a). The river basins with a gentle slope exhibited the highest TOCL. For the basins with moderate and gentle slopes, the TOCL increased from the small to medium scale and then decreased with further increases of spatial scales (Fig. 7b). Figure 7c shows the increases in TOCL along with scale in the rivers with medium Vc. We observed that basins with a high Vc presented increased TOCL from the small to medium scale and then decreased TOCL at the sizeable scale, whereas the basins with medium Vc presented increased TOCL from the small to large scale and then decreased TOCL to the great scale (Fig. 7c). In general, the TOCL increased along with the RD for all scales, whereas the TOCL in the sufficient RD basins increased along with the spatial scale (Fig. 7d).

## Discussion

### Control factors

The correlation analysis results (Fig. 2) demonstrate that the RD and the Rc have a substantial positive effect on the TOCL, which is consistent with the results of many studies^7^,^38^,^39^,^40^,^41^. This observation may have been related to the fluvial flow that occurs with water erosion, which transports topsoil and soil organic matter to the water channel, thereby increasing the organic carbon in river water^3^. The river water discharge was poorly correlated with the TSSL throughout China, which is similar to the finding that 10% of the world rivers account for over 60% of the sediment load^42^. Water discharge alone was not able to predict the sediment flux except in one region^43^. The Yellow River was believed to transport the highest sediment load among major rivers of the world because the Loess Plateau primarily contributes high sediment concentrations instead of water discharge^44^. When considering organic carbon concentrations, a dilution effect in which concentrations decrease along with discharge was observed among Chinese rivers. This result is consistent with studies in the Yangtze River^41^, the Yellow River^41^, the Yukon River^45^, and the Mississippi River^46^. We observed that the TSSC and the organic carbon concentration (DOCC and POCC) were strongly and positively correlated^47^,^48^,^49^; therefore, the TSSC also exhibited a dilution effect, which decreased along with the RD.

Climate factors have an important influence on river organic carbon. The organic carbon concentrations (DOCC and POCC) decreased with the MAP and MAT, although the organic carbon loads (DOCL and POCL) increased primarily with the MAP and MAT. This result may have been caused by the increased discharge observed in the high MAP and MAT regions in China, which dilutes the concentration^41^. The Rc clearly increased with the MAT and MAP, although a negative MAT-Rc relationship was observed in Mutema’s global meta-analysis^23^. The primary reason for this inconsistent result was because the MAT and MAP were strongly correlated with the continental monsoon climate across China. Although colder basins experienced lower evaporation, the scarcity of rainfall resulted in limited discharge. Our CART analysis indicated that the MAP and MAT were the primary factors controlling the Rc across China, which confirms this analysis.

The percent of vegetation coverage significantly reduced both the TSSC and the TSSL, which demonstrated that China’s “Grain-to-Green Program”^50^ is an effective method of conserving soil and water. The Yellow River Basin, which passes through the Loess Plateau, experienced the highest river sediment concentrations and fluxes^11^,^51^. Decreases in both the sediment concentration and flux of Yellow River Basin have been observed in recent years, which is primarily because of vegetation restoration^8^. With increases in Vc, the runoff also increased, although the TSSL decreased. This result was most likely because of the significant decrease in the TSSC without a clear increase in the runoff with higher Vc. At certain catchment scales, vegetation conservation reduced the declining trend in total sediment yield because of a decline in runoff 52. Interestingly, the TOCL increased along with the vegetation coverage. This result may have been caused by the increase in runoff and ecosystem production along with increases in vegetation coverage, which resulted in an increase in the TOCL. Using the CART approach, we observed that Vc was one of the primary factors controlling the TSSC, TSSL, and TOCL.

The TSSL and TOCL were greatly reduced in the high RSCI rivers because of small or large reservoirs^43^. With the CART technique, we also observed that the RSCI was one of the primary factors controlling the TSSC, TSSL, and TOCL. Dams and reservoirs trap total suspended sediments and organic carbon because of the large bulk of impounded water^5^,^27^,^28^,^47^, which results in a higher RSCI and lower TSSL and TOCL (Figs 2,6a and 7a). The impoundment of reservoir might contribute as an important carbon sink of terrestrial ecosystem^28^. The influence of dams or reservoirs on the TSS and the TOC may continue for many years because of increases in the demand for hydroelectric energy in China^25^. Our results in imply that human activities, such as reservoir or dam construction and vegetation coverage restoration, may have a more significant influence than climate (MAT and MAP) on the sediment and carbon transport in Chinese rivers. Although a previous global-scale meta-analysis did not observe the same results (because of limited information)^23^, the implication is reasonable based on our multiple linear regression model results (Table 3), and a similar pattern has been found in many single-basin studies in China^5^,^8^,^28^,^41^,^47^.

A previous study reported the substantial effect of soil on sediments^23^. However, our analysis results showed a minor effect of the soil characteristics (SOC and BD) on the TSS and TOC, which may have been because of the limited soil data in our dataset. In addition, representing the average soil characteristics across basins using the available soil survey data is difficult.

### Spatial scale effects

One of the objectives of this synthesis was to demonstrate the spatial-scale variability of water, sediment, and carbon transport among Chinese rivers because assessing the transport effects along spatial scales is critical for understanding the underlying processes and mechanisms. The Rc ranged from 0.32 to 0.46 at all scales and did not showed a constant trend along spatial scales (Table 2, Fig. 4). However, the river basins were analysed according to different classes of control factors. For instance, the Rc generally increased from the small to large scale in the humid and moist river basins, which may have been related to the additional contributions from the groundwater or interflow to the water channel^23^. However, for the wet basins, the trend of Rc overall decreased along with the spatial scale (Fig. 4a), which is similar to the pattern found in a previous study^53^. This decrease in the Rc can be explained by the mediation of higher Rc with a good runoff yields and concentration conditions (more precipitation) by in-channel transport^54^, such as water resource consumption by agriculture and industry along the river downstream. The lowest mean Rc was observed in the largest scale basins (Table 2) and determined according to the lowest mean MAP (Supplementary Table S1) in the great-size basins.

Mutema^23^ observed a high sediment concentration in the arid zone, and our results, which showed soil and water loss in the semiarid region^55^ and limited water dilution, corroborate this finding. In the semiarid and scarcity RD basins, the TSSC clearly decreased from the medium scale to the sizeable scale and subsequently increased to the great scale. This observation may have been related to the limited data for medium-sized basins and the high TSSC of certain medium-sized rivers, such as the Jinghe River, the Laohahe River, the Sanggan River, and the Yanghe River^56^, which increased the mean value of TSSC in the medium size rivers. However, this pattern must be verified with additional information. The river basins with many reservoirs (RSCI > 50%) increased the TSSC substantially along with scale (Fig. 5b), which can be explained as follows: the discharge and the RSCI presented a negative correlation (Fig. 2) and large rivers generally have more reservoirs^32^; and the Rc presented a decreasing trend along with scales in rivers with many reservoirs. Therefore, less water increases the TSSC, and this effect was enhanced along with spatial scale. The steep and small-sized river basins experienced a higher TSSC, which is consistent with the results of a previous meta-analysis^23^. Studies have indicated that a large decrease of organic matter is caused by water erosion on steep slopes^57^. The TSSC of the low Vc basins increased along with scale, suggesting an enhanced effect of soil erosion in the less vegetated zones along with scale.

TSSL significantly decreased from small (768 g·m^−2^·a^−1^) to medium spatial scale basins (258 g·m^−2^·a^−1^), which is different from Mutema’s results^23^. The reduced mean slope gradient from small to larger area may lead to less erosion, more deposition, and thus lower sediment flux^10^. This suggest that vegetation restoration in small basin is an effective way to conserve water and soil. The small rivers with high TSSLs were primarily observed in north China, such as the Kuyehe River, the Wudinghe River, and the Zulihe River^51^. The wind-deposited Loess Plateau with high silt content and fine particles of loessial soil was believed one of highest erodibility region in the world^58^, which is part of the reason for the high sediment load in this region. Small rivers in northern China generally experience lower temperatures and less precipitation (the r_s_ of the MAP-latitude and MAT-latitude are −0.83 and −0.86, respectively; Fig. 2) as well as serious soil and water loss issues, which is observed in the small rivers of the middle reach of the Yellow River Basin^51^. However, with increases in the basin area, the redeposition of entrained sediments may cause a decrease in the sediment load^23^. Mutema^23^ found a low sediment load in the arid zone because of limited water erosion. However, our results indicated that the small semiarid basins had a higher TSSL (Fig. 6d). This observation is because many small rivers in less rainfall regions has severe soil and water loss in China^59^. The great-sized river basins had the highest RSCI (Supplementary Table S1), and the RSCI was negatively correlated with the TSSL (Fig. 2), which explains the decreasing trend of the TSSL from the sizeable to great scale. For instance, the Three Gorges Project, which is located in the largest river in China (i.e., Yangtze River), is the largest hydropower project in the world^25^ and captures a substantial amount of sediment^5^,^28^. Many hydropower plants are still under construction or have been planned^25^; therefore, the influence of RSCI may continue for many years. The TSSL was reduced in the basin sizes with less water resources (e.g., scarcity RD and semiarid basins) and rivers with low vegetation coverage within China, which indicates that small-scale rivers are more vulnerable to erosion among the most fragile environmental types, particularly for the least vegetated (Vc < 20%) river basins.

The TOCL was affected by scale in the gentle-slope basins, and it decreased from the medium to the largest spatial scale (Fig. 7b), which is consistent with the results of a previous study^23^. This finding may be associated with the reduction of the Rc along with scale in the gentle-slope basins (Fig. 5c), which indicates that a reduction in discharge results in a reduction of TOC transport. The TOCL increased along with spatial scale in the sufficient discharge basins, which may be a result of the positive relationship between the annual average discharge (QA) and the basin size (r_s_ of 0.81, Fig. 2). The positive correlation between the amount of discharge and the organic carbon load is related to better erosive conditions^22^. Although fewer reservoir rivers showed increases of TOCL along with scales, this result may have been caused by limited data (Table 2, Fig. 7a); thus, it must be verified with additional research.

## Conclusions

A quantitative synthesis was performed to explore a dataset containing information on the transport of water, sediment and carbon within Chinese rivers, and this paper has summarized several of the associated conclusions. The MAP and MAT were the primary factors controlling the Rc. Human activities, such as dam construction and vegetation coverage restoration, significantly decreased the TSSL and the TOCL. However, the organic carbon concentration (POCC and DOCC) increased along with the RSCI. The Vc also significantly reduced the TSSC, and this result is related to soil and water conserve activities. The TSSC and the organic carbon concentration (POCC and DOCC) decreased along with the river water discharge because of a dilution effect, whereas the organic carbon load (POCL and DOCL) increased along with the river water discharge. However, the TSSL was poorly correlated with discharge. The TSS and the organic carbon variables (POC and DOC) were positively correlated. Our results indicate that complex patterns of water, sediments and carbon transport occur along spatial scales for different classes of environmental factors. The Rc increased from the small to large scale and subsequently decreased to the great scale except in wet, semiarid, and hot regions. The TSSC decreased from the small scale to the sizeable scale and subsequently increased to the great scale in semiarid regions. The TSSL generally decreased from the small to large scale, particularly in rivers with less water resources and vegetation coverage. The TOCL increased in basins with a low RSCI and a medium RSCI from the small to large scale. Our results present here will improve the understanding of water, sediment and carbon transport mechanism and contribute better water and land resource management and utilization strategies from different spatial scales. However, certain limitations occurred in this study. Extreme hydrologic and climatic events such as rainfall intensity and peak discharge, which have a significant influence on river transports^60^, were not included because of data limitations. Numerous hydrological, geological, biological, and chemical processes and the interactions of these processes may have altered the river transport processes. Therefore, future work on river water, sediment, and carbon delivery should include a more comprehensive investigation that integrates additional hydrological drivers.

## Additional Information

**How to cite this article**: Song, C. *et al*. Control factors and scale analysis of annual river water, sediments and carbon transport in China. *Sci. Rep*. **6**, 25963; doi: 10.1038/srep25963 (2016).

## Supplementary Material

Supplementary Information

Supplementary Table S1

## Figures and Tables

**Table 1:** Description of carbon variables and natural and anthropogenic factors.

Abbreviation	Name and unit	Description
Size	Size (km^2^)	watershed, catchment or basin area in km^2^
L	Length (km)	river or basin length or characteristic slope length
RD	Runoff depth (mm)	total annual runoff divided by basin area in mm
QA	Annual average discharge (m^3^ s^−1^)	annual average water discharge divided by time (seconds)
Rc	Runoff coefficient	runoff depth divided by annual rainfall Rc = RD/total rainfall
Rainfall	Total rainfall (mm)	precipitation received during the period of trial, i.e., annual precipitation
MAP	Mean annual precipitation (mm)	long-term mean annual precipitation - average precipitation over 30 years or more
MAT	Mean annual temperature (°C)	long-term mean annual temperature - average temperature over 30 years or more
S	Slope (%)	average slope gradient of the river basin
LAT	Latitude (°)	latitudinal position of river or catchment/basin outlet
LONG	Longitude (°)	longitudinal position of river or catchment/basin outlet
Vc	Vegetation coverage (%)	average vegetation coverage percent of the basin; when not provided, this value was calculated from the available NDVI: coverage(%) = −4.337–3.733*NDVI + 161.968*NDVI*NDVI^61^
RSCI	Reservoir storage capacity index (%)	the ratio of the total reservoir water storage capacity to the annual average water discharge of the contributing catchment
SOC	Soil organic carbon (%)	average soil organic carbon content of the basin; when SOM is indicated instead of SOC, then SOC = 0.58*SOM^26^,^62^
BD	Bulk density (g cm^−3^)	average soil bulk density; when not provided, this value was calculated as SOC: BD = −1.229ln(SOC) + 1.2901(for SOC < 6%) and BD = 1.3774e^(−0.0413SOC)^(for SOC >6%)^26^
TSSC	Total suspended sediment concentration (mg L^−1^)	average total suspended sediment concentration in the runoff
TSSL	Total suspended sediment load (g·m^−2^·a^−1^)	total suspended sediment load of unit area; when not provided, this value was calculated as TSSL = TSSC × RD ÷ 1000
POCC	Particulate organic carbon concentration (mg L^−1^)	average concentration of particulate organic carbon in the runoff
POCL	Particulate organic carbon load (g·m^−2^·a^−1^)	calculated or provided annual particulate organic carbon export unit area; when not provided, this value was calculated as POCL = POCC × RD ÷ 1000, where 1000 is the unit for conversion to a constant
DOCC	Dissolved organic carbon concentration (mg L^−1^)	average concentration of dissolved organic carbon in the runoff
DOCL	Dissolved organic carbon load (g·m^−2^·a^−1^)	calculated or given annual dissolved organic carbon export unit area; when not provided, this value was calculated as DOCL = DOCC × RD ÷ 1000, where 1000 is the unit for conversion to a constant
TOCC	Total organic carbon concentration (mg L^−1^)	average concentration of total organic carbon in the runoff
TOCL	Total organic carbon load (g·m^−2^·a^−1^)	calculated or given total annual organic carbon export unit area, TOCL = POCL + DOCL

**Table 2:** Classification of the size, environmental variables and general statistics.

Item	Classification criteria	Class	Mean			
			Rc	TSSC (mg/L)	TSSL (g·m^−2^·a^−1^)	TOCL (g·m^−2^·a^−1^)
Size	<15,000	Small	0.346 (63)	3,239 (33)	768 (58)	1.93 (29)
	15,000–100,000	Medium	0.416 (71)	3,187 (58)	254 (63)	6.77 (21)
	100,000–350,000	Sizeable	0.334 (55)	843 (53)	271 (54)	1.94 (8)
	350,000–700,000	Large	0.361 (63)	1,523 (59)	237 (55)	3.70 (20)
	>700,000	Great	0.321 (62)	3,430 (55)	255 (56)	1.30 (16)
RD	<100	Scarcity	0.097 (69)	8,395 (57)	713 (66)	0.90 (21)
	100–300	Insufficient	0.273 (65)	1,092 (52)	266 (58)	2.67 (12)
	300–600	Enough	0.430 (99)	808 (80)	299 (94)	1.40 (24)
	>600	Sufficient	0.560 (81)	218 (69)	179 (68)	6.04 (37)
MAP	250–600	Semiarid	0.145 (84)	6,814 (71)	597 (80)	0.92 (20)
	600–850	Moist	0.294 (52)	1,519 (42)	360 (48)	2.92 (9)
	850–1,500	Humid	0.453 (107)	664 (85)	283 (102)	1.32 (23)
	>1,500	Wet	0.512 (71)	182 (60)	160 (56)	5.56 (42)
MAT	<10	Cool	0.224 (88)	3,575 (53)	608 (83)	1.85 (13)
	10–20	Warm	0.380 (154)	2,534 (136)	311 (145)	1.58 (29)
	>20	Hot	0.473 (72)	190 (49)	123 (58)	4.59 (52)
S	<1	Gentle	0.336 (133)	2,214 (44)	200 (118)	3.65 (49)
	1–2	Moderate	0.393 (102)	1,343 (94)	273 (96)	5.01 (17)
	>2	Steep	0.349 (79)	5,071 (44)	735 (72)	1.589 (28)
RSCI	<10%	Low RSCI	0.450 (58)	1,431 (58)	414 (58)	2.69 (5)
	10–50%	Medium RSCI	0.457 (41)	314 (35)	116 (38)	2.41 (9)
	>50%	High RSCI	0.246 (45)	2,152 (39)	119 (40)	0.65 (12)
Vc	<20%	Low Vc	0.270 (32)	2,761 (24)	1,001 (30)	0.52 (4)
	20–40%	Medium Vc	0.376 (47)	723 (43)	166 (41)	3.05 (19)
	>40%	High Vc	0.331(22)	151 (17)	52 (17)	8.64 (7)

The sample size is provided in brackets. For the general statistics of the variables at different scales, see Supplementary Table S1.

**Table 3:** Multiple linear regression models.

Response variable	Component	Estimate	Std. Error	t value	Pr (>|t|)	Multiple R^2^	p-value	Degrees of freedom
Rc	(Intercept)	0.0994783	0.0182923	5.438	<0.001***	0.5445	<0.001	311
	MAT	−0.0082886	0.0022667	−3.657	<0.001 ***			
	MAP	0.0003681	0.0000272	13.532	<0.001 ***			
TSSC	(Intercept)	1612.56517	906.46327	1.779	0.0805	0.4025	<0.001	58
	MAP	−0.27744	1.30623	−0.212	0.8325			
	RSCI	20.06351	5.02247	3.995	<0.001 ***			
	RD	0.02242	1.95068	0.011	0.9909			
	S	34.27221	388.65927	0.088	0.9300			
	Vc	−46.17586	14.56646	−3.17	0.0024 **			
TSSL	(Intercept)	323.9008	58.1214	5.573	<0.001***	0.3205	<0.001	60
	RSCI	−0.8753	0.354	−2.473	0.0162 *			
	RD	0.3294	0.1302	2.53	0.0140 *			
	MAP	−0.2555	0.1325	−1.929	0.0585			
	MAT	5.4893	6.9371	0.791	0.4319			
	Vc	−3.0994	0.9683	−3.201	0.0022 **			
TOCL	(Intercept)	−9.003674	3.679632	−2.447	0.0308 *	0.6782	0.0056	12
	RSCI	0.021688	0.02108	1.029	0.3238			
	Vc	0.324286	0.119063	2.724	0.0185 *			
	S	0.462308	2.248804	0.206	0.8406			
	RD	−0.002305	0.006908	−0.334	0.7444			

The multiple linear regression results were based on the following formulas for the response variables: Rc = −0.00829 × MAT + 0.00037 × MAP + 0.0995 (R^2^ = 0.5445, P < 0.001); TSSC = −0.2774 × MAP + 20.06 × RSCI + 0.02 × RD + 34.27 × S - 46.18 × Vc + 1612 (R^2^ = 0.4025, P < 0.001); TSSL = −0.8753 × RSCI + 0.3294 × RD - 0.2555 × MAP + 5.4893 × MAT - 3.0994 × Vc + 323.9 (R^2^ = 0.3205, P < 0.001); and TOCL = 0.022 × RSCI + 0.324 × Vc + 0.462 × S - 0.0023 × RD - 9 (R^2^ = 0.6782, P = 0.0056).
