# Supplementary material for: Control factors and scale analysis of annual river water, sediments and carbon transport in China
Source: Sci Rep. 2016 May 11;6:25963. doi: 10.1038/srep25963 (PMC4863175; doi:10.1038/srep25963)
Supplement: Supplementary Information [file srep25963-s1.pdf]

Supplementary Information

Control factors and scale analysis of annual river water, sediments and carbon transport in China

Chunlin Song[1, 2], Genxu Wang*[1], Xiangyang Sun[1], Ruiying Chang[1], Tianxu Mao[1, 2]

[1]*Institute of Mountain Hazards and Environment, Chinese Academy of Sciences, Chengdu, 610041, China*

[2]*University of Chinese Academy of Sciences, Beijing, 100049, China*

*Correspondence to: wanggx@imde.ac.cn(Genxu Wang).

	Mean(Sample Size)				
	Small	Medium	Sizeable	Large	Great
TSSC(mg/L)	3239(33)	3187(59)	843(53)	1523(59)	3430(55)
TSSL(g m^{-2} a^{-1})	768(58)	254(64)	271(54)	237(55)	254(62)
POCC(mg/L)	76.73(12)	15.55(19)	25.59(7)	7.94(18)	25.56(10)
POCL(g m^{-2} a^{-1})	0.88(26)	4.27(20)	1.49(7)	2.87(19)	1.07(16)
DOCC(mg/L)	4.49(11)	2.73(17)	3.95(5)	3.22(18)	3.01(9)
DOCL(g m^{-2} a^{-1})	1.20(25)	2.99(19)	1.01(5)	1.56(19)	0.34(14)
Rc	0.36(63)	0.46(72)	0.33(55)	0.39(65)	0.32(62)
Size (km^2)	5522(63)	47179(72)	172035(55)	466285(65)	1192080(62)
L (km)	181(63)	634(72)	1275(55)	2400(65)	5227(62)
RD (mm)	463(63)	670(72)	312(55)	461(65)	313(62)
QA (m^3/s)	62(63)	925(72)	1676(55)	6313(65)	14301(62)
MAP (mm)	1060.6(63)	1212.4(72)	846.4(55)	1047.1(65)	802.9(62)
MAT (℃)	15.8(63)	15.1(72)	10.8(55)	12.3(65)	11.9(62)
S (%)	9.34(50)	1.79(72)	1.26(55)	0.90(65)	1.01(62)
Vc (%)	33.20(11)	45.04(26)	23.30(20)	29.19(23)	27.45(24)
RSCI (%)	20.72(3)	46.74(19)	42.75(29)	27.38(35)	73.45(48)
SOC (%)	1.37(58)	1.06(11)	1.25(3)	0.97(3)	\
BD (g/cm^3)	1.27(58)	1.29(11)	1.27(3)	1.29(3)	\

Table S1. General statistics for the variables in different scales (Small, Medium, Sizeable, Large, Great). Sample size is given is brackets. Abbreviations of the variables as shows in Table 1.

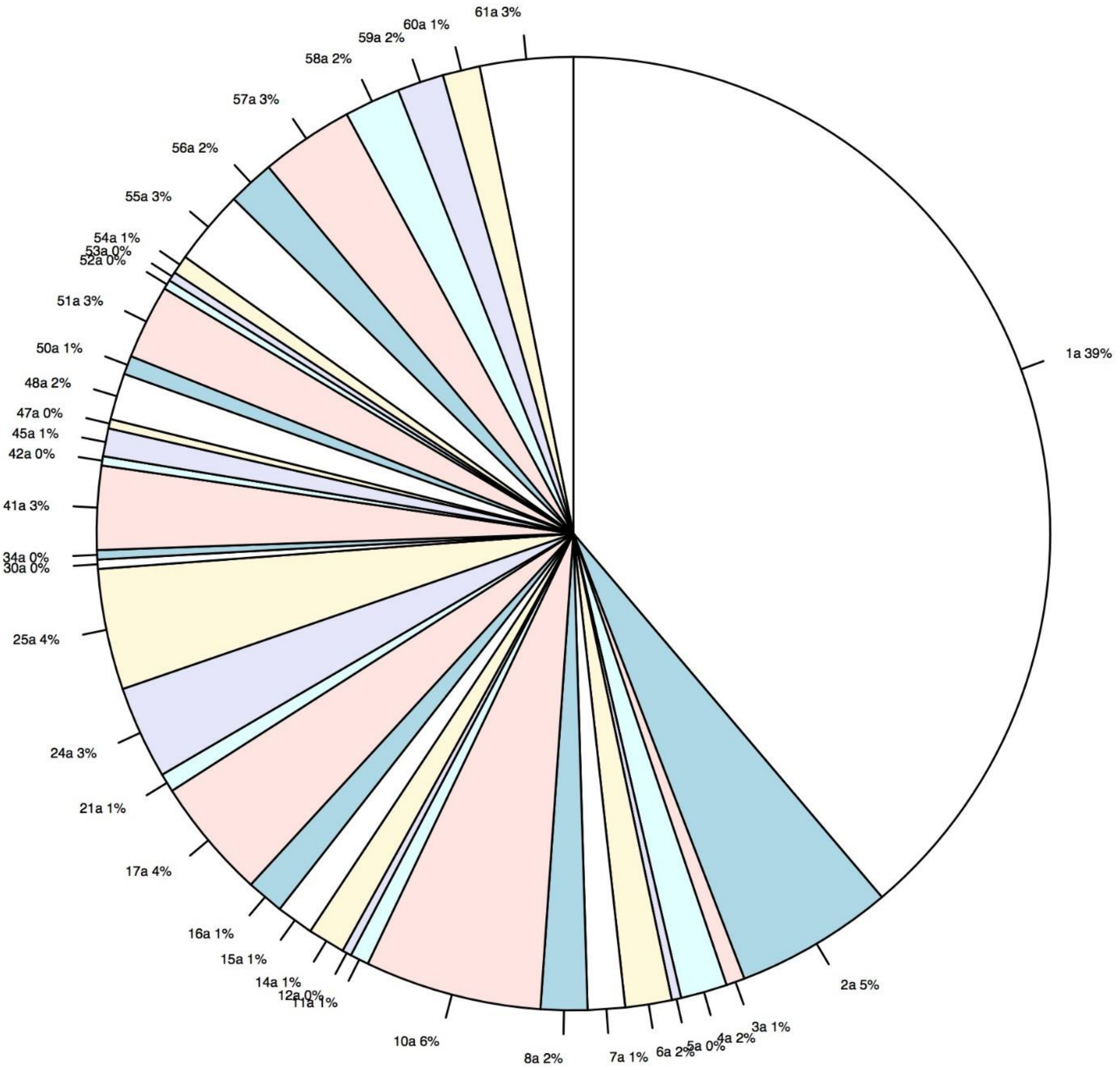

Figure S1. The age distribution of trials of our database.

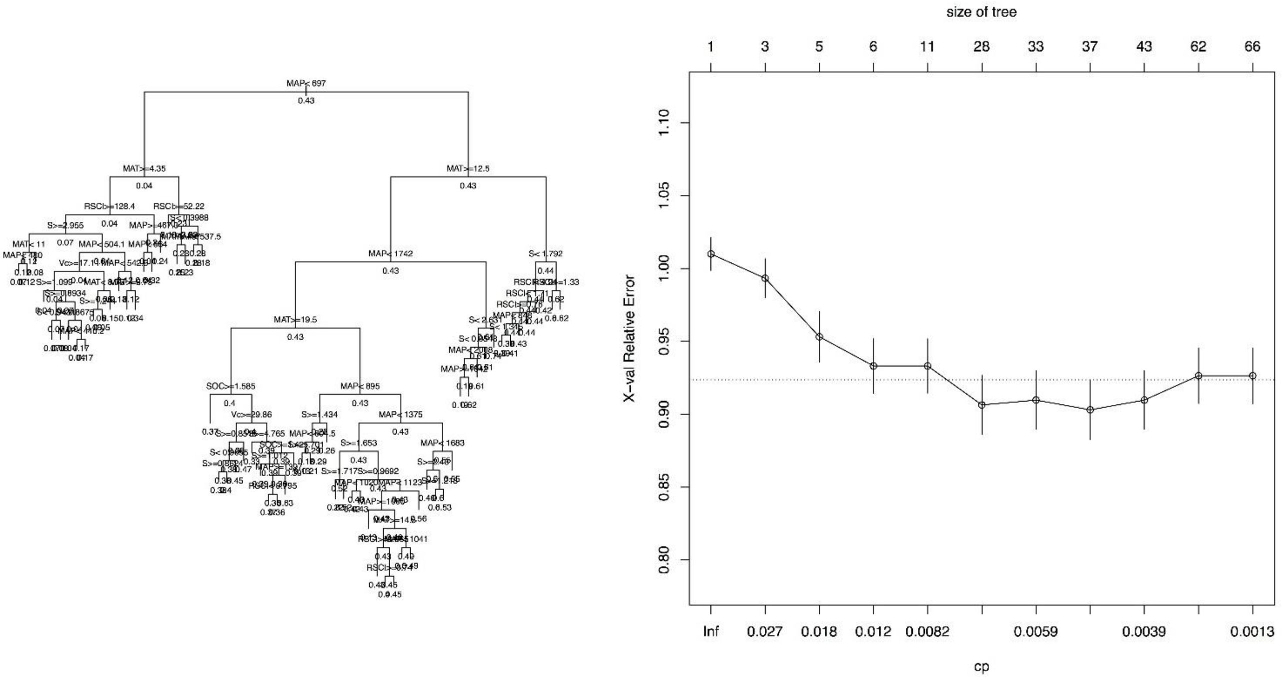

Figure S2. The left panel shows the relationships between Rc (runoff coefficient) and the environmental factors. The unpruned tree result was analysed via the CART (the classification and regression tree) analysis. Abbreviations of the variables as shows in Table 1. The right panel shows the tree size and relative error in the process of the CART (classification and regression tree) analysis of Rc. The above x-axis label was size of tree and the below x-axis label was cp value, while y-axis label was the value of relative error.

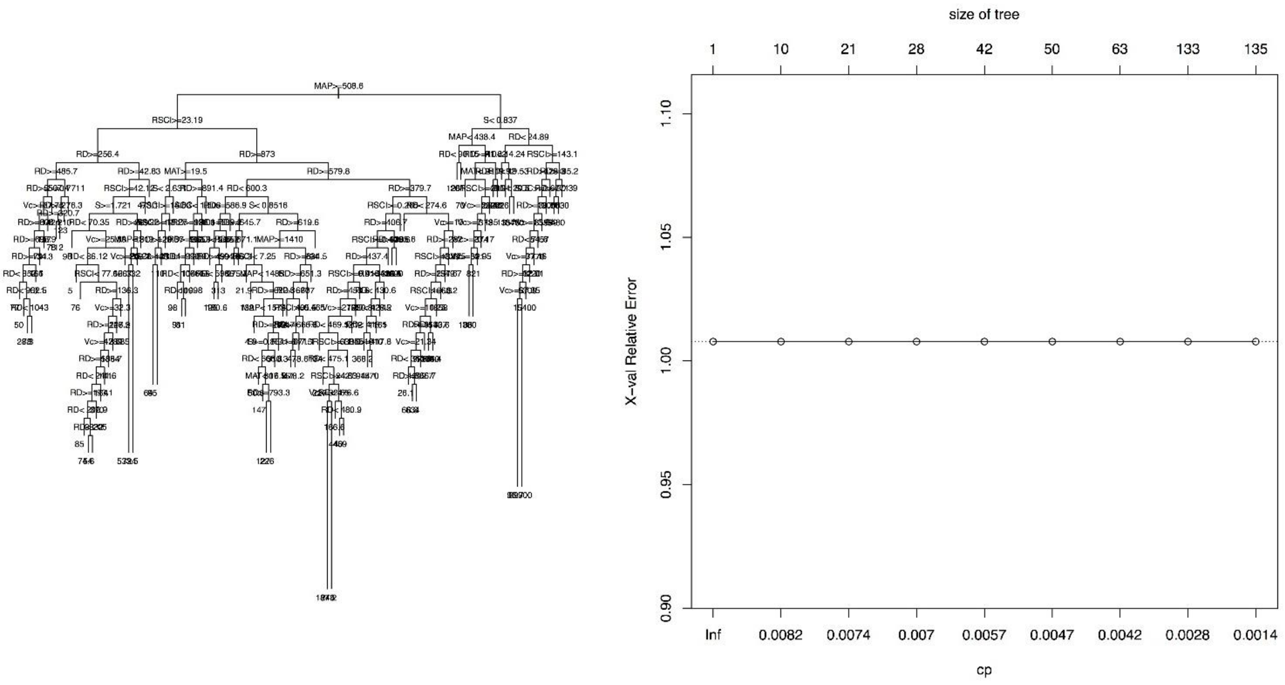

Figure S3. The left panel shows the relationships between TSSC (total suspended sediment concentration) and the environmental factors. The unpruned tree result was analysed via the CART (the classification and regression tree) analysis. Abbreviations of the variables as shows in Table 1. The right panel shows the tree size and relative error in the process of the CART (classification and regression tree) analysis of TSSC. The above x-axis label was size of tree and the below x-axis label was cp value, while y-axis label was the value of relative error.

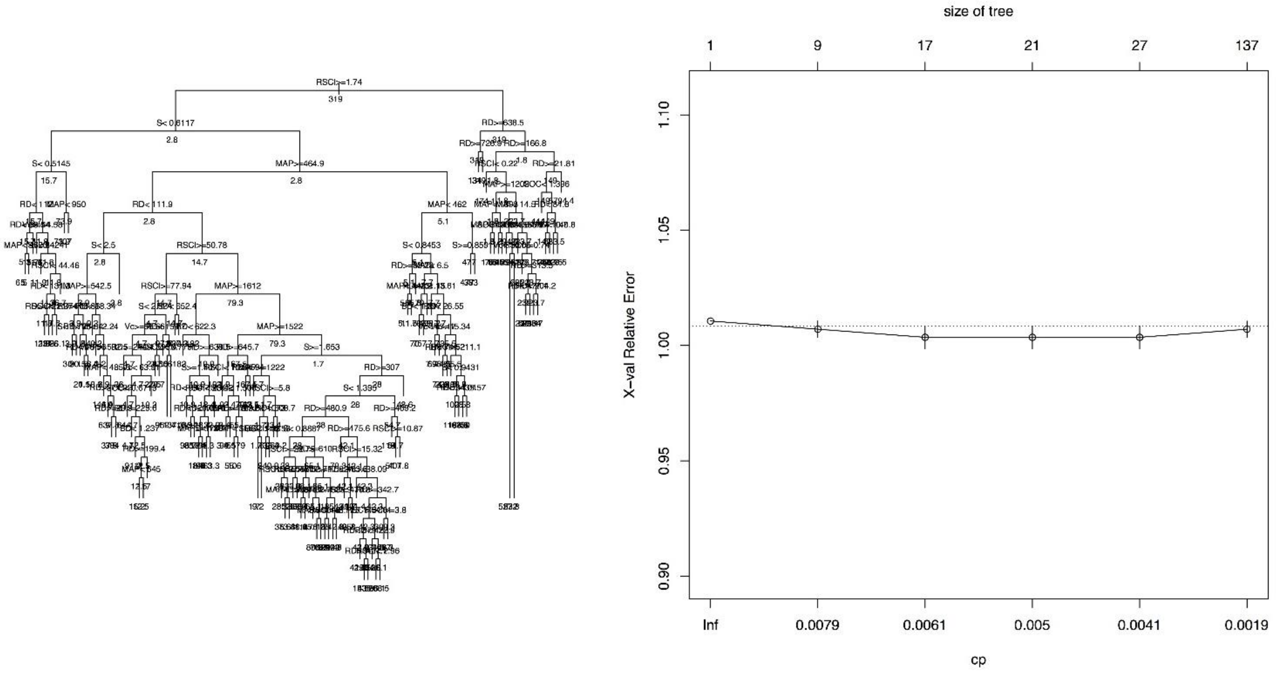

Figure S4. The left panel shows the relationships between TSSL (total suspended sediment load) and the environmental factors. The unpruned tree result was analysed via the CART (the classification and regression tree) analysis. Abbreviations of the variables as shows in Table 1. The right panel shows the tree size and relative error in the process of the CART (classification and regression tree) analysis of TSSL. The above x-axis label was size of tree and the below x-axis label was cp value, while y-axis label was the value of relative error.

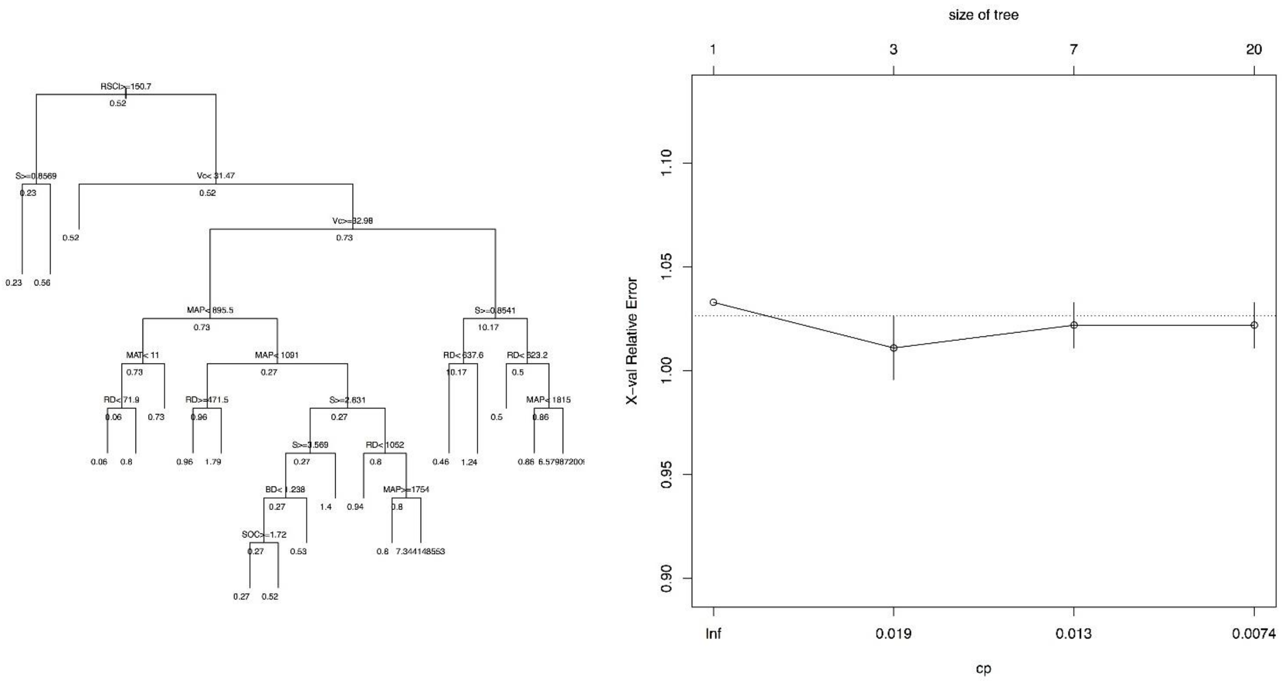

Figure S5. The left panel shows the relationships between TOCL (total organic carbon load) and the environmental factors. The unpruned tree result analysed via the CART (the classification and regression tree) analysis. Abbreviations of the variables as shows in Table 1. The right panel shows the tree size and relative error in the process of the CART (classification and regression tree) analysis of TOCL. The above x-axis label was size of tree and the below x-axis label was cp value, while y-axis label was the value of relative error.

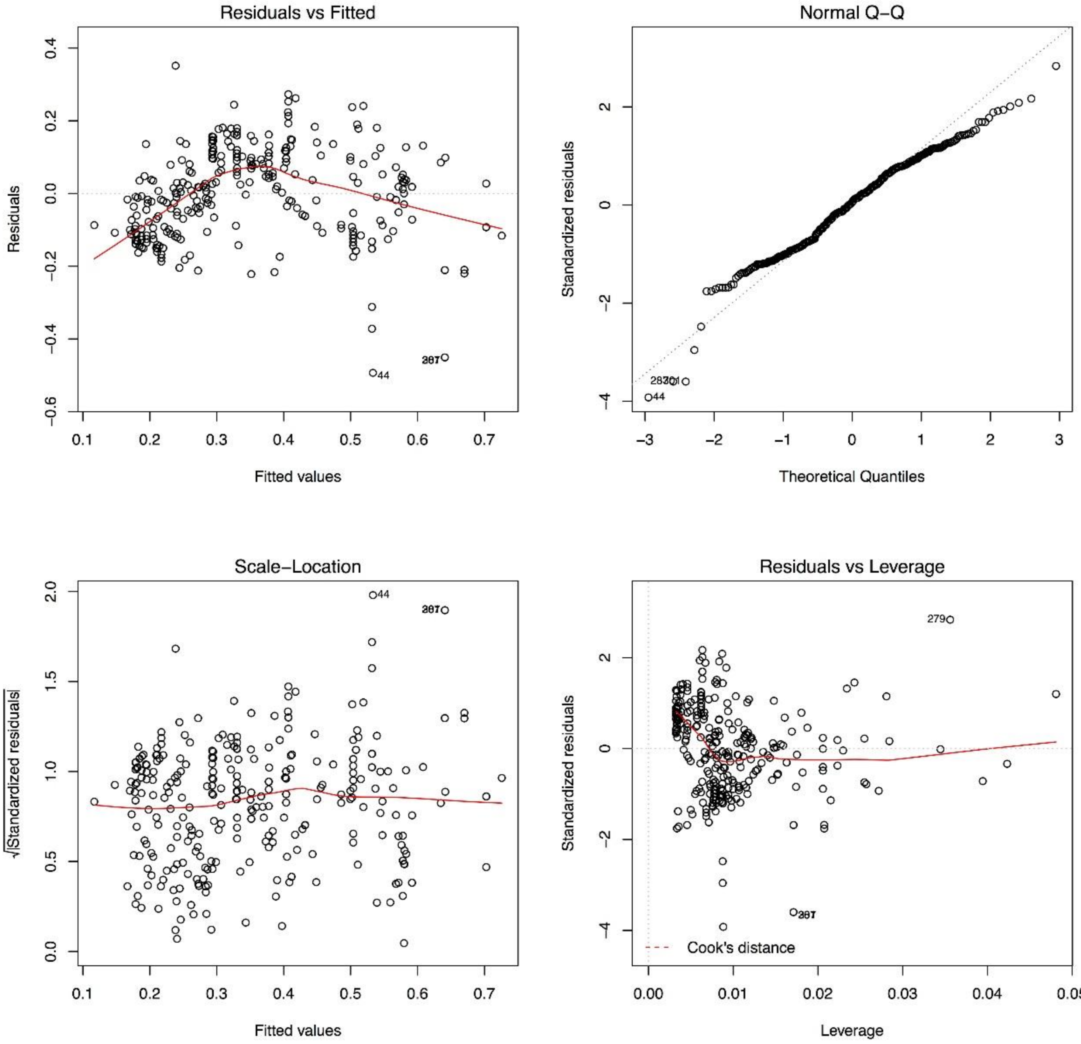

Figure S6. Diagnostic plots for the regression model of Rc on MAP and MAT.

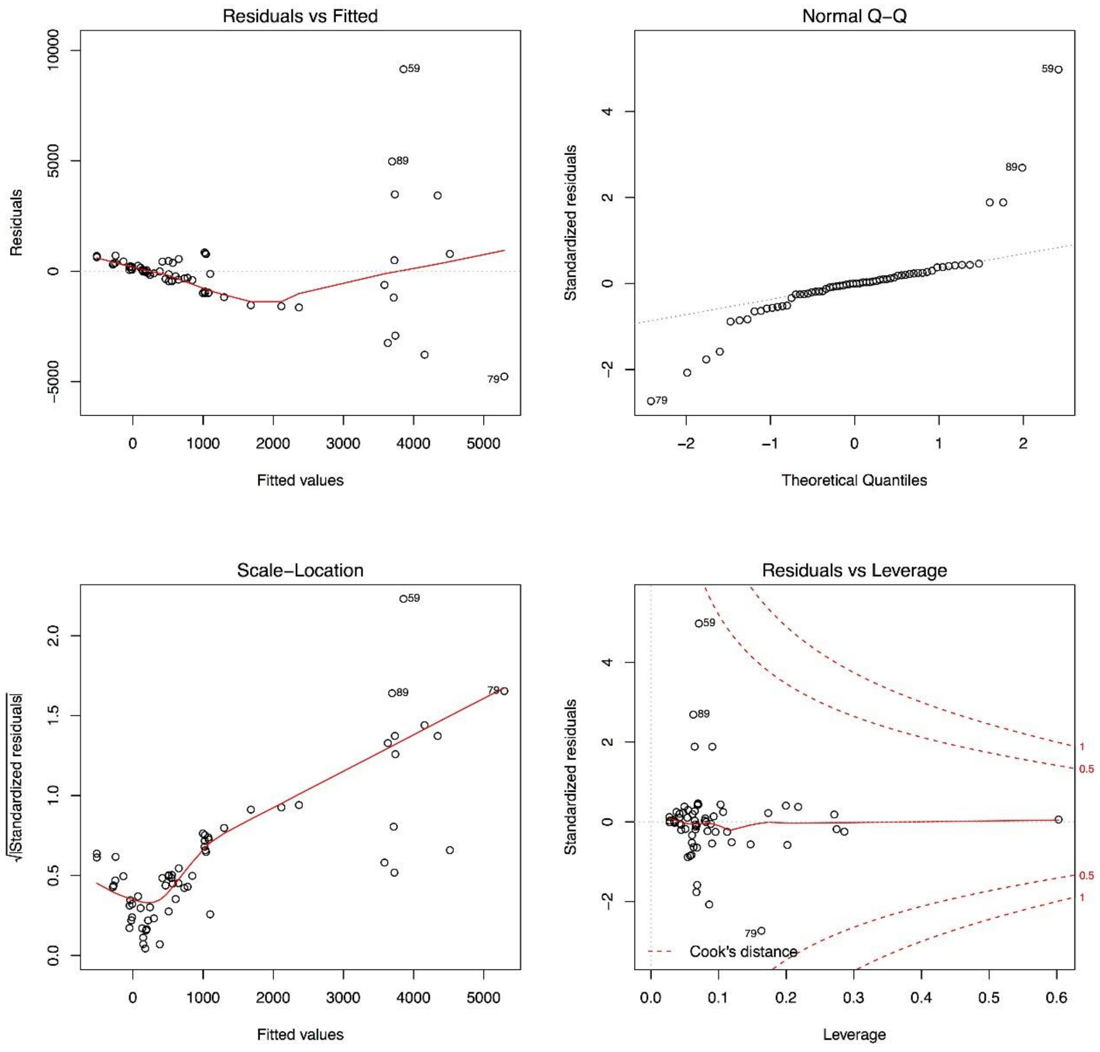

Figure S7. Diagnostic plots for the regression model of TSSC on MAP, RSCI, RD, S, and Vc.

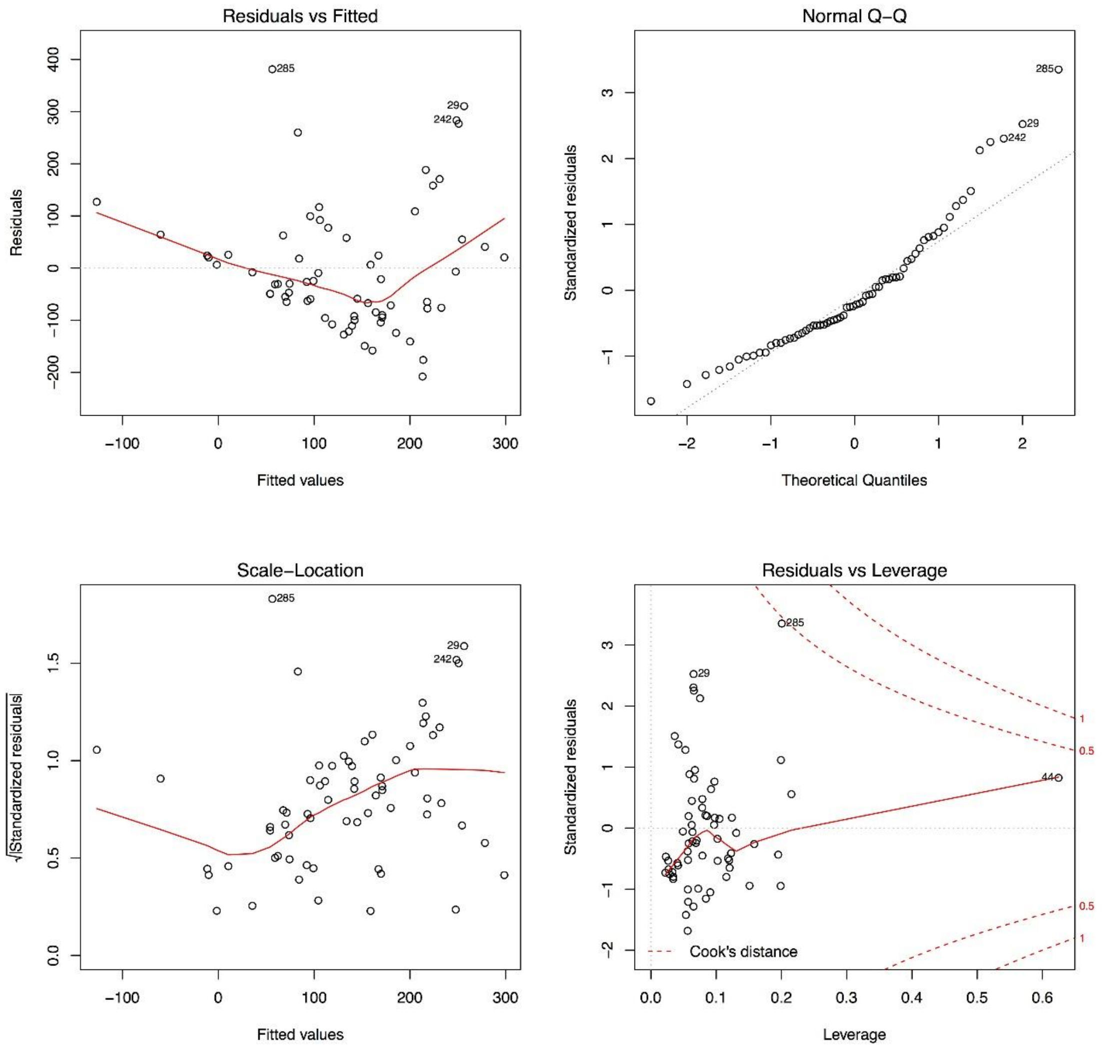

Figure S8. Diagnostic plots for the regression model of TSSL on RSCI, RD, MAP, MAT, and Vc.

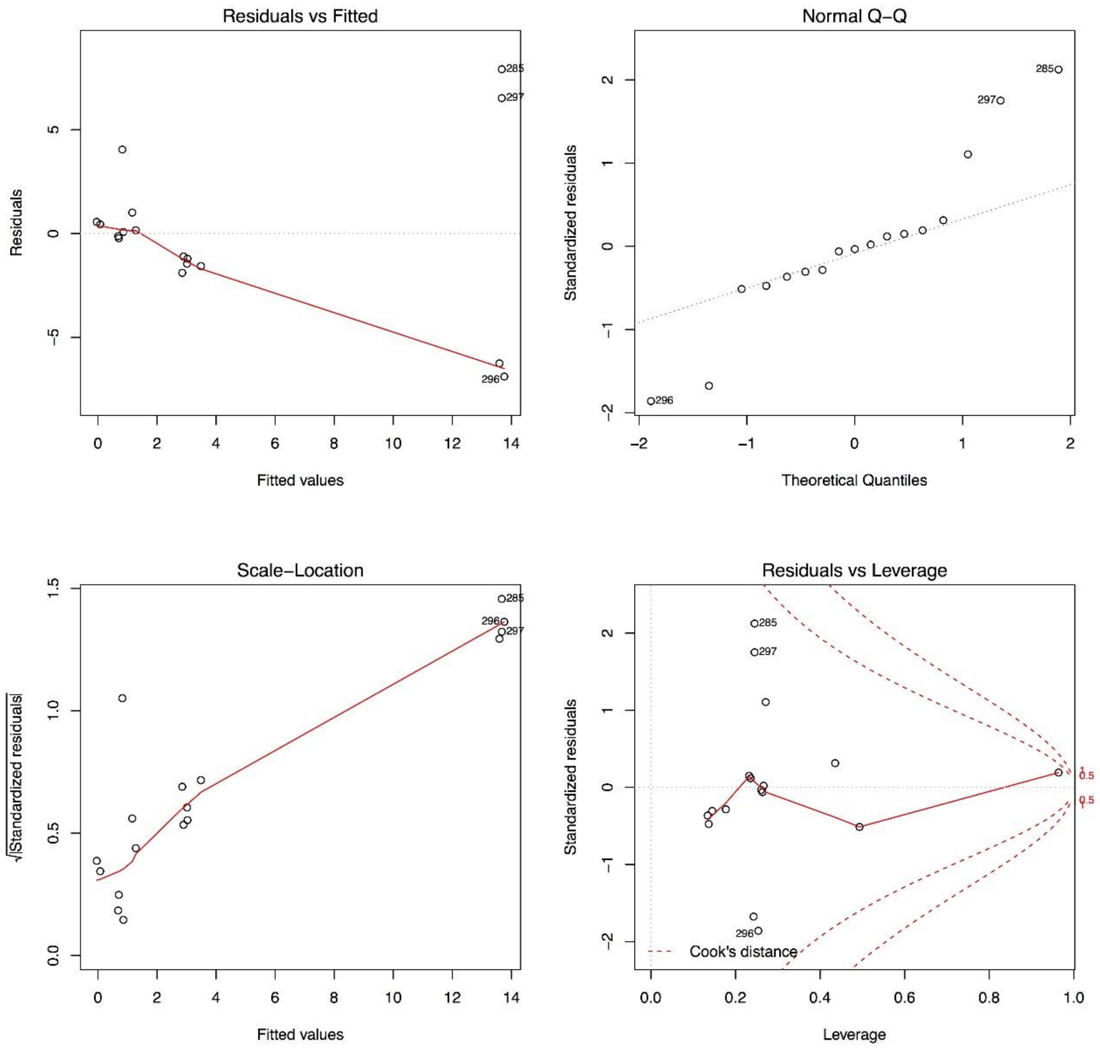

Figure S9. Diagnostic plots for the regression model of TOCL on RSCI, Vc, S, and RD.

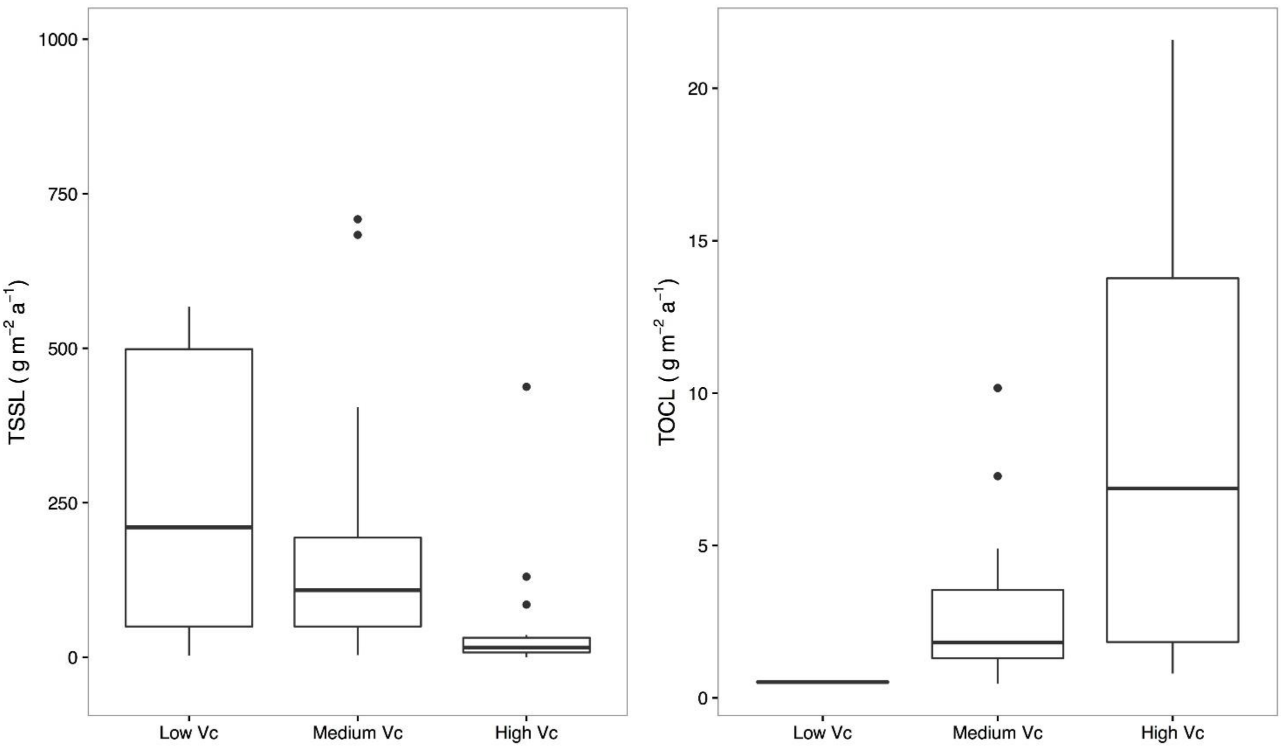

Figure S10. Boxplot of TSSL and TOCL in different classes of Vc.

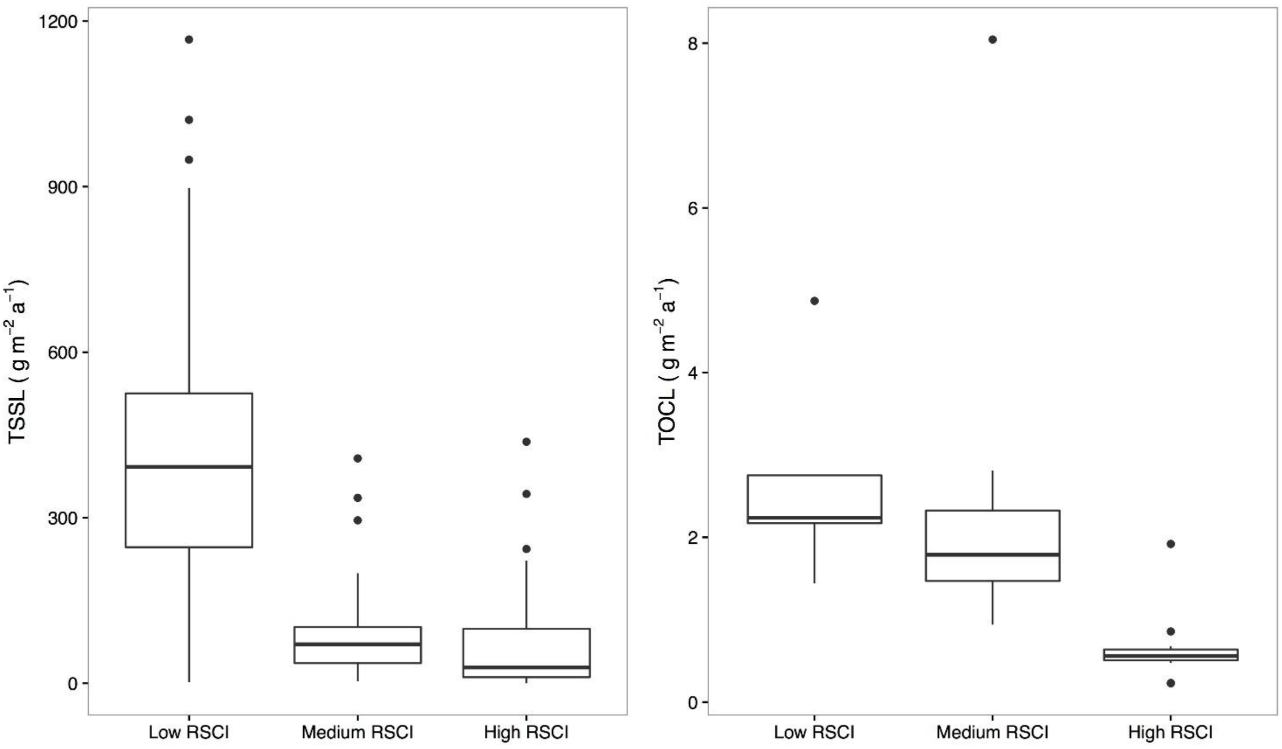

Figure S11. Boxplot of TSSL and TOCL in different classes of RSCI.

R codes:

```
##### install packages
install.packages(c("corrplot", "rpart", "mgcv", "ggplot2"))
library(corrplot,    rpart,    mgcv,    ggplot2)
setwd()

##### spearman's rank correlation analysis
cor.data <- read.csv("data.cor.csv", header=TRUE)
corr <- cor(cor.data, use="pairwise.complete.obs", method="spearman")
corr
pdf("corrplot.pdf", width = 15, height = 15, pointsize = 14)
corrplot.mixed(corr,    order="FPC",tl.pos="lt")    #correlation matrix plot
dev.off()    #save plot



##### CART
library(rpart)
set.seed(261212)
cart.data <- read.csv("data.cor.csv", header=TRUE)
attach(cart.data)
rc.cart <- rpart(Rc ~ MAP+MAT+S+Vc+RSCI+SOC+BD, cart.data, control=rpart.control(minsplit=8,cp=0.001),
method="class")
plot(rc.cart);text(rc.cart,all=TRUE,cex=.8)
printcp(rc.cart)
plotcp(rc.cart)
rc.cart.prune <- prune(rc.cart,cp=0.012)
plot(rc.cart.prune);text(rc.cart.prune, all=TRUE, cex=0.8)

set.seed(261213)
sc.cart <- rpart(TSSC ~ RD+MAP+MAT+Vc+S+RSCI+SOC+BD, cart.data,
control=rpart.control(minsplit=4,cp=0.001), method="class")
plot(sc.cart);text(sc.cart,cex=.8)
printcp(sc.cart)
plotcp(sc.cart)
sc.cart.prune <- prune(sc.cart,cp=0.0082); plot(sc.cart.prune); text(sc.cart.prune, all=TRUE, cex=0.8)

set.seed(261215)
sl.cart <- rpart(TSSL ~ RD+MAP+MAT+S+Vc+RSCI+SOC+BD, cart.data,
control=rpart.control(minsplit=4,cp=0.001), method="class")
plot(sl.cart);text(sl.cart,all=TRUE,cex=.8)
printcp(sl.cart)
plotcp(sl.cart)
sl.cart.prune <- prune(sl.cart,cp=0.0079); plot(sl.cart.prune, compress=FALSE, branch=1); text(sl.cart.prune,
```

```
all=TRUE, cex=0.8)

set.seed(261216)
set.seed(271702)
oc.cart <- rpart(TOCL ~ RD+MAP+MAT+S+Vc+RSCI+SOC+BD, cart.data,
control=rpart.control(minsplit=8,cp=0.005), method="class")
plot(oc.cart);text(oc.cart,all=TRUE,cex=.8)
printcp(oc.cart)
plotcp(oc.cart)
oc.cart.prune <- prune(oc.cart,cp=0.013)
plot(oc.cart.prune);text(oc.cart.prune, all=TRUE, cex=0.8)


pdf("cart.slscrcocl.pdf", width=20, height=18)
par(mfrow=c(2,2))
plot(rc.cart.prune, branch=1, margin=0.01, compress=FALSE); text(rc.cart.prune, all=TRUE, cex=1.5)
plot(sc.cart.prune, branch=1, margin=0.01, compress=FALSE); text(sc.cart.prune, all=TRUE, cex=1.5)
plot(sl.cart.prune, branch=1, margin=0.01, compress=FALSE); text(sl.cart.prune, all=TRUE, cex=1.5)
plot(oc.cart.prune, branch=1, margin=0.01, compress=FALSE); text(oc.cart.prune, all=TRUE, cex=1.5)
dev.off()


##### lm analysis

rc.lm <- lm(Rc ~ MAP+MAT)
summary(rc.lm)
pdf("rc.lm.pdf", width=10, height=10)
par(mfrow=c(2,2));plot(rc.lm)
dev.off()

sc.lm <- lm(TSSC ~ MAP+RSCI+RD+S+Vc)
summary(sc.lm)
pdf("sc.lm.pdf", width=10, height=10)
par(mfrow=c(2,2));plot(sc.lm)
dev.off()

sl.lm <- lm(TSSL ~ RSCI+RD+MAP+MAT+Vc)
summary(sl.lm)
pdf("sl.lm.pdf", width=10, height=10)
par(mfrow=c(2,2));plot(sl.lm)
dev.off()

oc.lm <- lm(TOCL ~ RSCI+Vc+S+RD)
summary(oc.lm)
```

```
pdf("oc.lm.pdf", width=10, height=10)
par(mfrow=c(2,2));plot(oc.lm)
dev.off()


##### Scale effects
### Rc plot

library(ggplot2)
rc.data <- read.csv("data.class.csv", header=TRUE)
rsci.class.data <- read.csv("data.class.rsci.csv", header=TRUE)
vc.class.data <- read.csv("data.class.vc.csv", header=TRUE)
rc.class.data <- read.csv("data.class.rc.csv", header=TRUE)

tapply(rc.data$Rc, list(rc.data$Size, rc.data$MAP), mean, na.rm=TRUE)
p1.1 <- ggplot(rc.data, aes(factor(Size),Rc)) +    geom_boxplot() + geom_point(aes(color=factor(MAP))) +
stat_summary(fun.y=mean, geom="line", aes(colour=factor(MAP), group=factor(MAP))) + guides(col =
guide_legend(ncol = 2))+ theme_classic()+theme(text = element_text(size=10), axis.text.x = element_text(angle=0,
vjust=1), legend.title = element_text(size=10), legend.title = element_text(size=10), legend.position=c(.8, .9)) +
ylim(0,1) + scale_x_discrete(labels=c("Small","Medium","Sizeable","Large","Great")) + labs(colour = "MAP",
x="Size", y="Rc") + scale_color_manual(values=c("1.Semiarid"="#F8766D", "2.Moist"="#A3A500",
"3.Humid"="#01B0F6", "4.Wet"="#00BF7D")) + annotate("text", x = 0.8, y = 1, label = "(a)")

tapply(rc.data$Rc, list(rc.data$Size, rc.data$MAT), mean, na.rm=TRUE)
p1.2 <- ggplot(rc.data, aes(factor(Size),Rc)) +    geom_boxplot() + geom_point(aes(color=factor(MAT))) +
stat_summary(fun.y=mean, geom="line", aes(colour=factor(MAT), group=factor(MAT))) + guides(col =
guide_legend(ncol = 2))+ theme_classic()+theme(text = element_text(size=10), axis.text.x = element_text(angle=0,
vjust=1), legend.title = element_text(size=10), legend.title = element_text(size=10), legend.position=c(.8, .9)) +
ylim(0,1) + scale_x_discrete(labels=c("Small","Medium","Sizeable","Large","Great"))    + labs(colour = "MAT",
x="Size", y="Rc") + scale_color_manual(values=c("1.Cool"="#00BF7D", "2.Warm"="#A3A500",
"3.Hot"="#F8766D")) + annotate("text", x = 0.8, y = 1, label = "(b)")


require(gridExtra)
pdf("Rcnew.pdf", width=8, height=3.33)
grid.arrange(p1.1, p1.2, ncol=2)
dev.off()


### TSSC plot
library(ggplot2)
rc.data <- read.csv("data.class.csv", header=TRUE)
rsci.class.data <- read.csv("data.class.rsci.csv", header=TRUE)
vc.class.data <- read.csv("data.class.vc.csv", header=TRUE)
```

```
rc.class.data <- read.csv("data.class.rc.csv", header=TRUE)

tapply(rc.data$SC, list(rc.data$Size, rc.data$RD), mean, na.rm=TRUE)
p2.0 <- ggplot(rc.class.data, aes(factor(Size),SC)) +    geom_boxplot() + geom_point(aes(color=factor(RD))) + stat_summary(fun.y=mean, geom="line", aes(colour=factor(RD), group=factor(RD))) + coord_cartesian(ylim =scales::expand_range(quantile(rc.data$SC, c(0.005, 0.9965), na.rm=TRUE),0.05))+guides(col = guide_legend(ncol = 2))+ theme_classic()+theme(text = element_text(size=10), axis.text.x = element_text(angle=0, vjust=1), legend.title = element_text(size =10), legend.title = element_text(size=10), legend.position=c(.7, .85)) + scale_x_discrete(labels=c("Small","Medium","Sizeable","Large","Great"))    + labs(colour = "Runoff depth", x="Size", y=bquote('TSSC ('*~mg~ L^-1*')')) + scale_color_manual(values=c("1.Scarcity"="#F8766D", "2.Insufficient"="#A3A500", "3.Enough"="#01B0F6", "4.Sufficient"="#00BF7D")) + annotate("text", x = 0.7, y = 40000, label = "(c)")


tapply(rc.data$SC, list(rc.data$Size, rc.data$MAP), mean, na.rm=TRUE)
p2.1 <- ggplot(rc.data, aes(factor(Size),SC)) +    geom_boxplot() + geom_point(aes(color=factor(MAP))) +stat_summary(fun.y=mean, geom="line", aes(colour=factor(MAP), group=factor(MAP))) + guides(col = guide_legend(ncol = 2))+ theme_classic()+theme(text = element_text(size=10), axis.text.x = element_text(angle=0, vjust=1), legend.title = element_text(size =10), legend.title = element_text(size=10), legend.position=c(.8, .85))+ coord_cartesian(ylim =scales::expand_range(quantile(rc.data$SC, c(0.005, 0.9965), na.rm=TRUE),0.05)) + scale_x_discrete(labels=c("Small","Medium","Sizeable","Large","Great"))    + labs(colour = "MAP", x="Size", y=bquote('TSSC ('*~mg~ L^-1*')')) + scale_color_manual(values=c("1.Semiarid"="#F8766D", "2.Moist"="#A3A500", "3.Humid"="#01B0F6", "4.Wet"="#00BF7D")) + annotate("text", x = 0.7, y = 40000, label = "(a)")


tapply(rc.data$SC, list(rc.data$Size, rc.data$S), mean, na.rm=TRUE)
p2.2 <- ggplot(rc.data, aes(factor(Size),SC)) +    geom_boxplot() + geom_point(aes(color=factor(S))) + stat_summary(fun.y=mean, geom="line", aes(colour=factor(S), group=factor(S))) + theme_classic()+theme(text = element_text(size=10), axis.text.x = element_text(angle=0, vjust=1), legend.title = element_text(size=10), legend.title = element_text(size=10), legend.position=c(.8, .9))+ coord_cartesian(ylim =scales::expand_range(quantile(rc.data$SC, c(0.005, 0.9965), na.rm=TRUE),0.05)) + scale_x_discrete(labels=c("Small","Medium","Sizeable","Large","Great"))    + labs(colour = "Slope", x="Size", y=bquote('TSSC ('*~mg~ L^-1*')')) + scale_color_manual(values=c("1.Steep"="#A3A500", "2.Moderate"="#01B0F6", "3.Gentle"="#00BF7D")) + annotate("text", x = 0.7, y = 40000, label = "(d)")


tapply(rc.data$SC, list(rc.data$Size, rc.data$Vc), mean, na.rm=TRUE)
p2.3 <- ggplot(vc.class.data, aes(factor(Size),SC)) +    geom_boxplot() + geom_point(aes(color=factor(Vc))) + stat_summary(fun.y=mean, geom="line", aes(colour=factor(Vc), group=factor(Vc))) + theme_classic()+theme(text = element_text(size=10), axis.text.x = element_text(angle=0, vjust=1), legend.title = element_text(size =10), legend.title = element_text(size=10), legend.position=c(.8, .85))+ coord_cartesian(ylim =scales::expand_range(quantile(rc.data$SC, c(0.05, 0.95), na.rm=TRUE),0.05)) + scale_x_discrete(labels=c("Small","Medium","Sizeable","Large","Great"))    + labs(colour = "Vegetation
```

```
coverage", x="Size", y=bquote('TSSC ('*~mg~ L^-1*')')) + scale_color_manual(values=c("1.Low Vc"="#F8766D", "2.Medium Vc"="#A3A500", "3.High Vc"="#00BF7D"))+ annotate("text", x = 0.7, y = 14000, label = "(e)")


tapply(rc.data$SC, list(rc.data$Size, rc.data$RSCI), mean, na.rm=TRUE)
p2.4 <- ggplot(rsci.class.data, aes(factor(Size),SC)) +    geom_boxplot() + geom_point(aes(color=factor(RSCI))) + stat_summary(fun.y=mean, geom="line", aes(colour=factor(RSCI), group=factor(RSCI))) + theme_classic() +theme(text = element_text(size=10), axis.text.x = element_text(angle=0, vjust=1), legend.title = element_text(size =10), legend.title = element_text(size=10), legend.position=c(.4, .85))+ coord_cartesian(ylim =scales::expand_range(quantile(rc.data$SC, c(0.05, 0.95), na.rm=TRUE),0.05)) + scale_x_discrete(labels=c("Small","Medium","Sizeable","Large","Great"))    + labs(colour = "Reservoir storage\ncapacity index", x="Size", y=bquote('TSSC ('*~mg~ L^-1*')')) + scale_color_manual(values=c("1.Low RSCI"="#00BF7D", "2.Medium RSCI"="#A3A500", "3.High RSCI"="#01B0F6")) + annotate("text", x = 0.7, y = 14000, label = "(b)")

require(gridExtra)
pdf("SCnew.pdf", width=8, height=10)
grid.arrange(p2.1, p2.4, p2.0, p2.2, p2.3, ncol=2)
dev.off()


### TSSL plot
library(ggplot2)
rc.data <- read.csv("data.class.csv", header=TRUE)
rsci.class.data <- read.csv("data.class.rsci.csv", header=TRUE)
vc.class.data <- read.csv("data.class.vc.csv", header=TRUE)
rc.class.data <- read.csv("data.class.rc.csv", header=TRUE)

tapply(rc.data$SL, list(rc.data$Size, rc.data$MAP), mean, na.rm=TRUE)
p3.0 <- ggplot(rc.data, aes(factor(Size),SL)) +    geom_boxplot() + geom_point(aes(color=factor(MAP))) + stat_summary(fun.y=mean, geom="line", aes(colour=factor(MAP), group=factor(MAP))) + guides(col = guide_legend(ncol = 2)) + theme_classic()+theme(text = element_text(size=10), axis.text.x = element_text(angle=0, vjust=1), legend.title = element_text(size=10), legend.title = element_text(size=10), legend.position=c(.8, .85)) + coord_cartesian(ylim =scales::expand_range(quantile(rc.data$SL, c(0.006, 0.994), na.rm=TRUE),0.05)) + scale_x_discrete(labels=c("Small","Medium","Sizeable","Large","Great"))    + labs(colour = "MAP", x="Size", y=bquote('TSSL ('*~g~ m^-2~a^-1*')')) + scale_color_manual(values=c("1.Semiarid"="#F8766D", "2.Moist"="#A3A500", "3.Humid"="#01B0F6", "4.Wet"="#00BF7D")) + annotate("text", x = 0.7, y = 6000, label = "(d)")


tapply(rc.data$SL, list(rc.data$Size, rc.data$Vc), mean, na.rm=TRUE)
p3.1 <- ggplot(vc.class.data, aes(factor(Size),SL)) +    geom_boxplot() + geom_point(aes(color=factor(Vc))) + stat_summary(fun.y=mean, geom="line", aes(colour=factor(Vc), group=factor(Vc))) + theme_classic()+theme(text
```

```
= element_text(size=10), axis.text.x = element_text(angle=0, vjust=1), legend.title = element_text(size =10), legend.title = element_text(size=10), legend.position=c(.8, .85)) + coord_cartesian(ylim =scales::expand_range(quantile(rc.data$SL, c(0.01, 0.992), na.rm=TRUE),0.05)) + scale_x_discrete(labels=c("Small","Medium","Sizeable","Large","Great"))    + labs(colour = "Vegetation coverage", x="Size", y=bquote('TSSL ('*~g~ m^-2~a^-1*')')) + scale_color_manual(values=c("1.Low Vc"="#F8766D", "2.Medium Vc"="#A3A500", "3.High Vc"="#00BF7D")) + annotate("text", x = 0.7, y = 5000, label = "(e)")

tapply(rc.data$SL, list(rc.data$Size, rc.data$RSCI), mean, na.rm=TRUE)
p3.2 <- ggplot(rsci.class.data, aes(factor(Size),SL)) +    geom_boxplot() + geom_point(aes(color=factor(RSCI))) + stat_summary(fun.y=mean, geom="line", aes(colour=factor(RSCI), group=factor(RSCI))) + theme_classic() +theme(text = element_text(size=10), axis.text.x = element_text(angle=0, vjust=1), legend.title = element_text(size =10), legend.title = element_text(size=10), legend.position=c(.8, .8))+ coord_cartesian(ylim =scales::expand_range(quantile(rc.data$SL, c(0.02, 0.98), na.rm=TRUE),0.05)) + scale_x_discrete(labels=c("Small","Medium","Sizeable","Large","Great"))    + labs(colour = "Reservoir storage\ncapacity index", x="Size", y=bquote('TSSL ('*~g~ m^-2~a^-1*')')) + scale_color_manual(values=c("1.Low RSCI"="#00BF7D", "2.Medium RSCI"="#A3A500", "3.High RSCI"="#01B0F6")) + annotate("text", x = 0.7, y = 1400, label = "(a)")

tapply(rc.data$SL, list(rc.data$Size, rc.data$RD), mean, na.rm=TRUE)
p3.3 <- ggplot(rc.data, aes(factor(Size),SL)) +    geom_boxplot() + geom_point(aes(color=factor(RD))) + stat_summary(fun.y=mean, geom="line", aes(colour=factor(RD), group=factor(RD))) + guides(col = guide_legend(ncol = 2))+ theme_classic()+theme(text = element_text(size=10), axis.text.x = element_text(angle=0, vjust=1), legend.title = element_text(size =10), legend.title = element_text(size=10), legend.position=c(.75, .85))+ coord_cartesian(ylim =scales::expand_range(quantile(rc.data$SL, c(0.01, 0.99), na.rm=TRUE),0.05)) + scale_x_discrete(labels=c("Small","Medium","Sizeable","Large","Great"))    + labs(colour = "Runoff depth", x="Size", y=bquote('TSSL ('*~g~ m^-2~a^-1*')')) + scale_color_manual(values=c("1.Scarcity"="#F8766D", "2.Insufficient"="#A3A500", "3.Enough"="#01B0F6", "4.Sufficient"="#00BF7D")) + annotate("text", x = 0.7, y = 3000, label = "(b)")

tapply(rc.data$SL, list(rc.data$Size, rc.data$MAT), mean, na.rm=TRUE)
p3.4 <- ggplot(rc.data, aes(factor(Size),SL)) +    geom_boxplot() + geom_point(aes(color=factor(MAT))) + stat_summary(fun.y=mean, geom="line", aes(colour=factor(MAT), group=factor(MAT))) + guides(col = guide_legend(ncol = 2))+ theme_classic()+theme(text = element_text(size=10), axis.text.x = element_text(angle=0, vjust=1), legend.title = element_text(size =10), legend.title = element_text(size=10), legend.position=c(.8, .9))+ coord_cartesian(ylim =scales::expand_range(quantile(rc.data$SL, c(0.01, 0.99), na.rm=TRUE),0.05)) + scale_x_discrete(labels=c("Small","Medium","Sizeable","Large","Great"))    + labs(colour = "MAT", x="Size", y=bquote('TSSL ('*~g~ m^-2~a^-1*')')) + scale_color_manual(values=c("1.Cool"="#00BF7D", "2.Warm"="#A3A500", "3.Hot"="#F8766D")) + annotate("text", x = 0.7, y = 3000, label = "(c)")
```

```
require(gridExtra)
pdf("SLnew.pdf", width=8, height=10)
grid.arrange(p3.2, p3.3, p3.4, p3.0, p3.1, ncol=2)
dev.off()


### TOCL plot
library(ggplot2)
rc.data <- read.csv("data.class.csv", header=TRUE)
rsci.class.data <- read.csv("data.class.rsci.csv", header=TRUE)
vc.class.data <- read.csv("data.class.vc.csv", header=TRUE)
rc.class.data <- read.csv("data.class.rc.csv", header=TRUE)

tapply(rc.data$TOCL, list(rc.data$Size, rc.data$RD), mean, na.rm=TRUE)
p4.0 <- ggplot(rc.data, aes(factor(Size),TOCL)) +    geom_boxplot() + geom_point(aes(color=factor(RD))) +
stat_summary(fun.y=mean, geom="line", aes(colour=factor(RD), group=factor(RD))) + guides(col =
guide_legend(ncol = 1))+ theme_classic()+theme(text = element_text(size=10), axis.text.x = element_text(angle=0,
vjust=1), legend.title = element_text(size =10), legend.title = element_text(size=10), legend.position=c(.8, .85))+
scale_x_discrete(labels=c("Small","Medium","Sizeable","Large","Great"))    + labs(colour = "Runoff depth",
x="Size", y=bquote('TOCL ('*~g~ m^-2~a^-1*')')) + ylim(0,25) +
scale_color_manual(values=c("1.Scarcity"="#F8766D", "2.Insufficient"="#A3A500", "3.Enough"="#01B0F6",
"4.Sufficient"="#00BF7D"))    + annotate("text", x = 0.7, y = 25, label = "(d)")

tapply(rc.data$TOCL, list(rc.data$Size, rc.data$S), mean, na.rm=TRUE)
p4.1 <- ggplot(rc.data, aes(factor(Size),TOCL)) +    geom_boxplot() + geom_point(aes(color=factor(S))) +
stat_summary(fun.y=mean, geom="line", aes(colour=factor(S), group=factor(S))) + theme_classic()+theme(text =
element_text(size=10), axis.text.x = element_text(angle=0, vjust=1), legend.title = element_text(size =10),
legend.title = element_text(size=10), legend.position=c(.85, .85)) +
scale_x_discrete(labels=c("Small","Medium","Sizeable","Large","Great"))    + labs(colour = "Slope", x="Size",
y=bquote('TOCL ('*~g~ m^-2~a^-1*')')) + ylim(0,25) + scale_color_manual(values=c("1.Steep"="#A3A500",
"2.Moderate"="#01B0F6", "3.Gentle"="#00BF7D"))    + annotate("text", x = 0.7, y = 25, label = "(b)")


tapply(rc.data$TOCL, list(rc.data$Size, rc.data$Vc), mean, na.rm=TRUE)
p4.2 <- ggplot(vc.class.data, aes(factor(Size),TOCL)) +    geom_boxplot() + geom_point(aes(color=factor(Vc))) +
stat_summary(fun.y=mean, geom="line", aes(colour=factor(Vc), group=factor(Vc))) + theme_classic()+theme(text
= element_text(size=10), axis.text.x = element_text(angle=0, vjust=1), legend.title = element_text(size =10),
legend.title = element_text(size=10), legend.position=c(.85, .85)) +
scale_x_discrete(labels=c("Small","Medium","Sizeable","Large","Great"))    + labs(colour =
"Vegetation\ncoverage", x="Size", y=bquote('TOCL ('*~g~ m^-2~a^-1*')')) + ylim(0,25) +
scale_color_manual(values=c("1.Low Vc"="#F8766D", "2.Medium Vc"="#A3A500", "3.High Vc"="#00BF7D"))
+ annotate("text", x = 0.7, y = 25, label = "(c)")
```

```
tapply(rc.data$TOCL, list(rc.data$Size, rc.data$RSCI), mean, na.rm=TRUE)
p4.3 <- ggplot(rsci.class.data, aes(factor(Size),TOCL)) +    geom_boxplot() +
geom_point(aes(color=factor(RSCI))) + stat_summary(fun.y=mean, geom="line", aes(colour=factor(RSCI),
group=factor(RSCI))) + theme_classic()+theme(text = element_text(size=10), axis.text.x = element_text(angle=0,
vjust=1), legend.title = element_text(size =10), legend.title = element_text(size=10), legend.position=c(.8, .85))+
ylim(0,15) + scale_x_discrete(labels=c("Small","Medium","Sizeable","Large","Great"))    + labs(colour =
"Reservoir storage\ncapacity index", x="Size", y=bquote('TOCL ('*~g~ m^-2~a^-1*')')) +
scale_color_manual(values=c("1.Low RSCI"="#00BF7D", "2.Medium RSCI"="#A3A500", "3.High
RSCI"="#01B0F6")) + annotate("text", x = 0.7, y = 15, label = "(a)")

require(gridExtra)
pdf("TOCLnew.pdf", width=8, height=6.6)
grid.arrange(p4.3, p4.1, p4.2, p4.0)
dev.off()

##### Pie chart
yrs <- read.csv("yrs.csv", header = TRUE)
label <- paste(yrs$Year)
label <- paste(label,"a",sep="")
label <- paste(label, round(yrs$Count/sum(yrs$Count)*100))
label <- paste(label,"%",sep="")
pie(yrs$Count, labels=label, radius = 1, clockwise = TRUE, cex=0.3)
```
